# Variation in Intraoperative Opioid Administration by Patient, Clinician, and Hospital Contribution

**DOI:** 10.1001/jamanetworkopen.2023.51689

**Published:** 2024-01-16

**Authors:** Michael L. Burns, Paul Hilliard, John Vandervest, Graciela Mentz, Ace Josifoski, Jomy Varghese, Clark Fisher, Sachin Kheterpal, Nirav Shah, Mark C. Bicket

**Affiliations:** 1 Department of Anesthesiology, University of Michigan Medical School, Ann Arbor; 2 Department of Anesthesiology, Yale School of Medicine, New Haven, Connecticut; 3 Opioid Prescribing Engagement Network, Institute for Healthcare Innovation and Policy, University of Michigan, Ann Arbor

## Abstract

**Importance:**

The opioid crisis has led to scrutiny of opioid exposures before and after surgical procedures. However, the extent of intraoperative opioid variation and the sources and contributing factors associated with it are unclear.

**Objective:**

To analyze attributable variance of intraoperative opioid administration for patient-, clinician-, and hospital-level factors across surgical and analgesic categories.

**Design, Setting, and Participants:**

This cohort study was conducted using electronic health record data collected from a national quality collaborative database. The cohort consisted of 1 011 268 surgical procedures at 46 hospitals across the US involving 2911 anesthesiologists, 2291 surgeons, and 8 surgical and 4 analgesic categories. Patients without ambulatory opioid prescriptions or use history undergoing an elective surgical procedure between January 1, 2014, and September 11, 2020, were included. Data were analyzed from January 2022 to July 2023.

**Main Outcomes and Measures:**

The rate of intraoperative opioid administration as a continuous measure of oral morphine equivalents (OMEs) normalized to patient weight and case duration was assessed. Attributable variance was estimated in a hierarchical structure using patient, clinician, and hospital levels and adjusted intraclass correlations (ICCs).

**Results:**

Among 1 011 268 surgical procedures (mean [SD] age of patients, 55.9 [16.2] years; 604 057 surgical procedures among females [59.7%]), the mean (SD) rate of intraoperative opioid administration was 0.3 [0.2] OME/kg/h. Together, clinician and hospital levels contributed to 20% or more of variability in intraoperative opioid administration across all analgesic and surgical categories (adjusting for surgical or analgesic category, ICCs ranged from 0.57-0.79 for the patient, 0.04-0.22 for the anesthesiologist, and 0.09-0.26 for the hospital, with the lowest ICC combination 0.21 for anesthesiologist and hosptial [0.12 for the anesthesiologist and 0.09 for the hospital for opioid only]). Comparing the 95th and fifth percentiles of opioid administration, variation was 3.3-fold among anesthesiologists (surgical category range, 2.7-fold to 7.7-fold), 4.3-fold among surgeons (surgical category range, 3.4-fold to 8.0-fold), and 2.2-fold among hospitals (surgical category range, 2.2-fold to 4.3-fold). When adjusted for patient and surgical characteristics, mean (square error mean) administration was highest for cardiac surgical procedures (0.54 [0.56-0.52 OME/kg/h]) and lowest for orthopedic knee surgical procedures (0.19 [0.17-0.21 OME/kg/h]). Peripheral and neuraxial analgesic techniques were associated with reduced administration in orthopedic hip (51.6% [95% CI, 51.4%-51.8%] and 60.7% [95% CI, 60.5%-60.9%] reductions, respectively) and knee (48.3% [95% CI, 48.0%-48.5%] and 60.9% [95% CI, 60.7%-61.1%] reductions, respectively) surgical procedures, but reduction was less substantial in other surgical categories (mean [SD] reduction, 13.3% [8.8%] for peripheral and 17.6% [9.9%] for neuraxial techniques).

**Conclusions and Relevance:**

In this cohort study, clinician-, hospital-, and patient-level factors had important contributions to substantial variation of opioid administrations during surgical procedures. These findings suggest the need for a broadened focus across multiple factors when developing and implementing opioid-reducing strategies in collaborative quality-improvement programs.

## Key Points

**Question:**

How much intraoperative variability in opioid administration is attributable to the patient, clinician, and hospital?

**Findings:**

In this cohort study of 1 011 268 surgical procedures at 46 hospitals performed by 2911 anesthesiologists and 2291 surgeons, clinicians and hospitals combined to contribute 20% or more of intraoperative opioid administration variation using adjusted intraclass correlations.

**Meaning:**

This study found substantial contributions to variation in intraoperative opioid administration at clinician, hospital, and patient levels, underscoring the need to consider these factors when evaluating opioid-reducing strategies.

## Teaser

This cohort study investigates the contributions of patients, clinicians, and hospitals to variation in intraoperative opioid administration for surgical procedures.

## Introduction

The long-standing public health crisis of overdose deaths in the US has prompted a reexamination of opioid uses, including in the context of surgical procedures.^1^ Opioids represent a key component of surgical pain management, but significant risks appear to accompany their use.^2^ Patients receiving opioid prescriptions after surgical procedures encounter significant risks postoperatively, ranging from readmissions and complications to unintentional prolonged opioid use or diversion to nonpatient individuals after surgical procedures.^3,4^ Length of stay, readmissions, and costs all increase in patients with vs without preoperative opioid use.^5^ While opioid use before and after surgical procedures has received much attention, opioid administration in the operating room has received less scrutiny despite its ubiquity, and there remains lack of understanding of its significance.

Clinician and hospital intraoperative opioid administrations are not well understood, and these variables are often omitted in perioperative enhancement initiatives, such as Enhanced Recovery After Surgery (ERAS) protocols. These protocols, as evidence-based care processes for patients who underwent surgical procedures, seek to reduce variation in perioperative care and are associated with improved clinical outcomes and costs.^6^ Pain control is only one of several outcomes considered, and resulting strategies use broad, multimodal analgesia techniques for opioid reduction, lacking focused approaches.^6,7,8,9^ Furthermore, intraoperative opioid-sparing regimens on which these efforts rely have been limited by small sample sizes or single procedural types,^10,11,12^ and extreme techniques, such as opioid-free analgesia, have questionable patient safety profiles.^13^ This results in knowledge gaps and controversies around optimal intraoperative opioid-administration strategies. The uncertainty has impeded efforts to align opioid use with actual patient needs, leaving perioperative clinicians to rely on heuristics, historical approaches, and incomplete protocols. The opportunity remains to leverage large amounts of electronic health record (EHR) data to develop a comprehensive understanding of practice patterns for opioid administration in the operating room,^14^ including multiple factors associated with their variation. Optimization of intraoperative opioid use may complement protocols intended to reduce perioperative patient risk and advance recovery.

To address these gaps, we analyzed intraoperative records using a nationwide registry and investigated differential contributions of patient, clinician (surgeon and anesthesiologist), and hospital characteristics for several common surgical procedures. We hypothesized that there exists an important contribution to the variance of intraoperative opioid administration beyond patient factors, with clinician and hospital factors contributing to the total variability of intraoperative opioid administration and that these contributions persist across different surgical and analgesic technique categories. These findings may identify a new avenue to improve the quality and safety of opioid use around surgical care.

## Methods

The Institutional Review Board at the University of Michigan exempted this cohort study from review and informed consent because it was a secondary analysis of deidentified data. This study follows the Strengthening the Reporting of Observational Studies in Epidemiology (STROBE) reporting guideline.

### Data Source

Data were extracted from the Multicenter Perioperative Outcomes Group (MPOG) registry, a clinical registry and national quality-improvement program consisting of academic and nonacademic hospitals contributing more than 20 million anesthesia records that represent diverse perioperative practice settings across the US.^15^ Within MPOG, perioperative records are automatically extracted from the local EHR and undergo quality checks to ensure the accuracy of patient demographics, medications, surgical procedures, and other characteristics relevant to intraoperative care.^16^ Data collection is audited quarterly with demonstration of high fidelity.^15^

### Study Cohort

This study included adult patients aged 18 years or older between January 1, 2014, and September 11, 2020, undergoing major (base unit values ≥6) surgical procedures within 8 relevant surgical categories grouped by anesthesiology Current Procedural Terminology codes: upper abdomen, lower abdomen, cardiac, hysterectomy, orthopedic hip, orthopedic knee, orthopedic spine, and vascular surgical procedures (eAppendix 1 and eTable 1 in Supplement 1). Defined as the clinician with the most time signed into each surgical procedure, a single anesthesiologist and surgeon were identified. Surgical procedures were excluded if conducted on the same patient less than 30 days apart or if missing data elements for independent variables (eAppendix 2, eFigure 1, and eTable 2 in Supplement 1). Additional exclusions were patient history of opioid, cocaine, amphetamine, antidepressant, psychedelic, or alcohol use based on *International Classification of Diseases, Ninth Revision *(*ICD-9*) and *International Statistical Classification of Diseases and Related Health Problems, Tenth Revision *(*ICD-10*) codes (eAppendix 3 and eTable 3 in Supplement 1) or based on prescription opioid medication listed in a preoperative or home medication list (eAppendix 4 in Supplement 1); patient aged less than 18 years; data missing to calculate opioid administration; low surgical procedure counts for anesthesiologists (<25 surgical procedures) or hospitals (<100 surgical procedures); and patients with an American Society of Anesthesiologists (ASA) physical status of 5, 6, missing, or unknown. The National Provider Identifier (NPI) linked anesthesiologists practicing at multiple hospitals. Because surgeons lacked NPIs, we treated each surgeon as a unique clinician and limited analyses to surgeons with 25 or more surgical procedures to minimize the potential of surgeon duplication. Analgesic categories consisted of neuraxial analgesia, peripheral nerve block, remifentanil, adjuvant analgesia, and opioid only (see eAppendix 5 and 6 in Supplement 1 for full category definitions). Categories were not mutually exclusive, except opioid only, which noted the absence of other techniques.

### Outcome

The primary outcome for this study was intraoperative opioid use, defined as the cumulative amount of all opioids administered to a patient during the intraoperative period in oral morphine equivalents (OMEs) normalized to patient weight in kilograms and case duration, yielding an outcome in units of OMEs per kilogram per hour. To calculate normalized OME, data were collected on all opioid medications administered during the intraoperative period, which spanned from documented start to end of anesthetic care. Opioid characteristics (name, dose, and route) were identified and standardized to OME based on previously published conversion factors (eAppendix 7 and eTable 4 in Supplement 1).^17,18,19,20,21,22,23,24,25,26^

### Independent Variables

Additional covariates were assessed. These included patient age, race, sex, body mass index (calculated as weight in kilograms divided by height in meters squared), ASA physical status (where 1 represents a healthy patient, 2 represents a patient with mild systemic disease, 3 represents a patient with severe systemic disease, and 4 represents a patient with severe systemic disease posing a constant threat to life), length of stay, case duration, number of Elixhauser comorbidities, and indicators for emergency status, weekend, and holiday surgical procedures. Race was identified by the database as 1 of 3 categorical options: non-Hispanic Black, non-Hispanic White, and other.

### Statistical Analysis

We used exploratory data analysis techniques to assess the distribution of outcome measures. Extreme values were identified using the Tukey fences approach and their removal from the analysis determined. Overall missing rates were less than 5%, thus complete case analysis results are presented. Potential unbalanced distribution on patient-level measures across surgical procedure categories was assessed using absolute standardized differences, with a value greater than 0.2 indicative of potential unbalance. The contribution to the variance of intraoperative opioid administration from patients, clinicians, and hospitals was assessed using intraclass correlation (ICC) estimates from a generalized linear mixed model (GLMM) with random intercept approach. The normal distribution was used with identity link and exchangeable correlation matrix. These models account for the natural hierarchical structure of the clinical data with patients nested within clinicians (anesthesiologist or surgeon) within hospitals. First, we used a null model to confirm the appropriateness of the multilevel model. We determined that ICCs greater than 0.01 are indicative of the importance of choosing a modeling strategy that addresses the multilevel structure of the data.^27,28^

ICCs were estimated across 2 primary clinicians: anesthesiologist and surgeon. Once the appropriateness of the 3-level GLMM was determined, it was used to test for differences in adjusted OME estimates across surgical categories using least square means. Adjusted ICCs were estimated using the same GLMM approach, adjusting for all independent variables (listed previously). Analyses were performed using SAS statistical software for Windows version 6.4 (SAS Institute) and used 2-tailed testing with *P* values < .05 considered significant. Data were analyzed from January 2022 to July 2023.

## Results

From an initial data set of 1 874 826 surgical operations, 1 011 268 surgical procedures (mean [SD] age of patients, 55.9 [16.2] years; 604 057 surgical procedures among females [59.7%]; 115 492 surgical procedures among Black patients [11.4%], 764 365 surgical procedures among White patients [75.6%], and 131 411 surgical procedures among patients with other race [13.0%]) (eAppendix 2 and eTable 2 in Supplement 1) met inclusion criteria. This analytical data set spanned 46 hospitals, 2911 anesthesiologists, and 2291 surgeons (eAppendix 2 and eFigure 1 in Supplement 1). The 3 most common surgical categories represented in this data set were 303 883 upper abdomen (30.0%), 256 715 lower abdomen(25.4%), and 187 448 orthopedic (spine) surgical procedures (18.5%). There were 277 680 outpatient surgical procedures (27.4%). Overall, patients were administered an opioid during the intraoperative period during 979 923 surgical procedures (96.9%). The mean (SD) level of intraoperative opioid use was 0.3 [0.2] OME/kg/h across all surgical procedures.

### Unadjusted Intraoperative Opioid Administration by Hospital and Clinician

**Figure 1.**
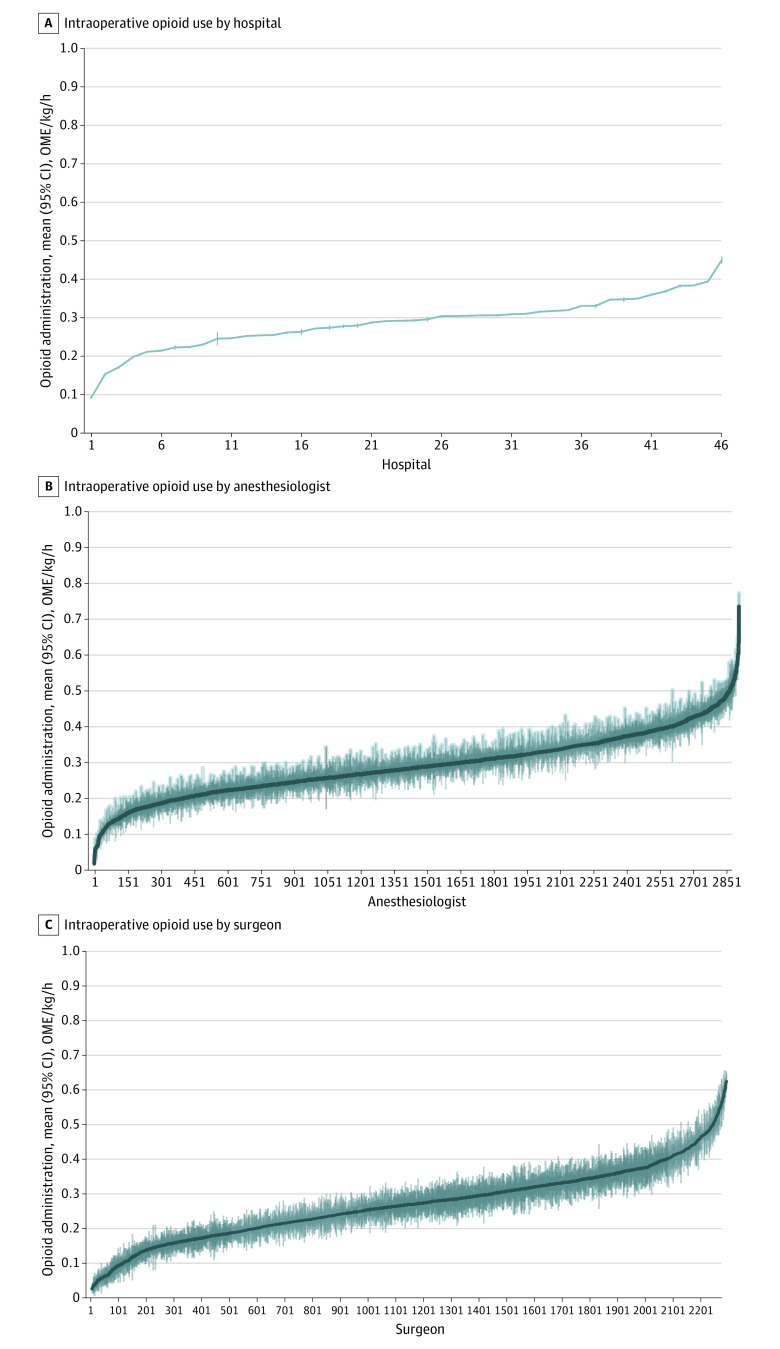
Intraoperative Opioid Administration by Hospital, Anesthesiologist, and Surgeon Intraoperative opioid administration is presented by hospital (A), anesthesiologist (B), and surgeon (C). Mean unadjusted intraoperative opioid administration is presented in oral morphine equivalents (OMEs) per kilogram per hour. Means were generated across all surgical and analgesic categories. Bars represent 95% CIs.

Unadjusted means of intraoperative opioid administration by hospital and surgical category can be found in eAppendix 8 and eFigure 2 in Supplement 1. The highest mean (SD) normalized OME by surgical category across all hospitals was cardiac surgical procedures (0.41 [0.12] OME/kg/h), and the lowest was orthopedic knee surgical procedures (0.17 [0.07] OME/kg/h). Intraoperative opioid administration varied by 5.0-fold across hospitals, with the mean administration in individual hospitals ranging from 0.09 OME/kg/h (95% CI, 0.09-0.09 OME/kg/h) to 0.45 OME/kg/h (95% CI, 0.44-0.46 OME/kg/h) (Table and Figure 1A). Hospital variation was highest for cardiac surgical procedures, which had an overall 8.9-fold variation, with mean administration for individual hospitals ranging from 0.07 OME/kg/h (95% CI, 0.07-0.07 OME/kg/h) to 0.60 OME/kg/h (95% CI, 0.58-0.61 OME/kg/h). Comparing the 95th percentile with the fifth percentile, hospital variation was 2.2-fold. In this comparison, the highest variation was found in orthopedic knee surgical procedures, a category exclusively comprising total knee arthroplasties, which varied 4.3-fold; mean administration for individual hospitals ranged from 0.05 OME/kg/h (95% CI, 0.04-0.05 OME/kg/h) to 0.32 OME/kg/h (95% CI, 0.29-0.34 OME/kg/h).

**Table.** Fold Variation Across Anesthesia, Clinician, and Hospital Factors^a^

Factor	Surgical category	No.	Fold variation overall^b^	Opioid administration, mean (95% CI), OME/kg/h		Fold variation by percentile	
				Minimum	Maximum	95th vs 5th	75th vs 25th
Hospital	All	46	5.0	0.09 (0.09-0.09)	0.45 (0.44-0.46)	2.2	1.3
	Upper abdomen	46	6.1	0.09 (0.08-0.09)	0.52 (0.51-0.53)	2.6	1.5
	Lower abdomen	46	6.7	0.08 (0.08-0.09)	0.55 (0.53-0.56)	2.4	1.3
	Cardiac	40	8.9	0.07 (0.07-0.07)	0.60 (0.58-0.61)	2.7	1.4
	Hysterectomy	46	6.7	0.07 (0.05-0.09)	0.47 (0.42-0.52)	2.9	1.7
	Hip	46	5.8	0.06 (0.06-0.07)	0.37 (0.36-0.39)	3.4	1.5
	Knee	46	6.9	0.05 (0.04-0.05)	0.32 (0.29-0.34)	4.3	1.6
	Spine	46	8.3	0.04 (0.04-0.05)	0.43 (0.41-0.46)	2.2	1.3
	Vascular	43	4.8	0.08 (0.08-0.08)	0.38 (0.33-0.42)	2.8	1.4
Anesthesiologist	All	2911	34.3	0.02 (0.01-0.03)	0.74 (0.70-0.77)	3.3	1.5
	Upper abdomen	2869	15.0	0.05 (0.00-0.09)	0.70 (0.60-0.79)	2.7	1.5
	Lower abdomen	2837	23.9	0.03 (0.01-0.05)	0.73 (0.65-0.81)	2.7	1.4
	Cardiac	909	36.3	0.23 (0.23-0.23)	0.84 (0.84-0.84)	7.4	2.1
	Hysterectomy	2233	50.2	0.02 (0.00-0.04)	0.84 (0.84-0.84)	4.2	1.6
	Hip	2117	208.2	0.00 (0.00-0.01)	0.85 (0.85-0.85)	7.2	1.9
	Knee	2110	345.5	0.00 (0.00-0.01)	0.85 (0.85-0.85)	7.7	1.9
	Spine	2697	16.3	0.02 (0.02-0.02)	0.77 (0.77-0.77)	3.3	1.6
	Vascular	1154	43.0	0.02 (0.00-0.04)	0.65 (0.65-0.65)	5.8	2.0
Surgeon	All	2291	23.0	0.03 (0.02-0.03)	0.62 (0.60-0.65)	4.3	1.7
	Upper abdomen	1433	344.4	0.00 (0.00-0.01)	0.78 (0.78-0.78)	4.5	1.7
	Lower abdomen	1576	42.8	0.02 (0.02-0.02)	0.64 (0.64-0.64)	3.4	1.6
	Cardiac	254	30.6	0.03 (0.03-0.03)	0.83 (0.83-0.83)	3.8	1.6
	Hysterectomy	555	35.3	0.02 (0.02-0.02)	0.85 (0.85-0.85)	4.7	1.8
	Hip	276	18.4	0.02 (0.00-0.06)	0.54 (0.54-0.54)	5.3	1.8
	Knee	275	14.3	0.03 (0.01-0.05)	0.52 (0.31-0.73)	6.0	2.3
	Spine	567	50.3	0.02 (0.00-0.03)	0.81 (0.81-0.81)	6.7	1.6
	Vascular	139	70.8	0.01 (0.01-0.01)	0.78 (0.78-0.78)	8.0	2.1

Abbreviation: OME, oral morphine equivalent.

^a^ Intraoperative opioid administration fold variation is given by hospital and clinician (surgeon or anesthesiologist), partitioned by surgical type. The minimum and maximum unadjusted intraoperative opioid administration levels are presented. Fold variations are represented overall and for 95th vs fifth and 75th vs 25th percentile.

^b^ Folds do not exactly represent minimum and maximum values from the table due to rounding; folds were calculated without rounding, while the table shows rounded minimum and maximum values.

The unadjusted mean of intraoperative opioid administration varied among anesthesiologists by 34.3-fold overall and 3.3-fold in the 95th vs fifth percentile comparison (Table and Figure 1B). Variation was highest among orthopedic knee surgical procedures, which had a 7.7-fold variation for the 95th vs fifth percentile, with mean administration for individuals ranging from less than 0.01 OME/kg/h (95% CI, <0.01 to <0.01 OME/kg/h) to 0.85 OME/kg/h (95% CI, 0.85 to 0.85 OME/kg/h). The lowest variation was for upper and lower abdomen surgical procedures, both with 95th vs fifth percentile variations of 2.7-fold. Surgeons varied by 23.0-fold, with 4.3-fold variation for the ninety-fifth vs fifth percentile. Mean individual surgeon unadjusted administrations ranged from 0.03 OME/kg/h (95% CI, 0.02 to 0.03 OME/kg/h) to 0.62 OME/kg/h (95% CI, 0.60 to 0.65 OME/kg/h) (Figure 1C). Variation by surgeon was highest among vascular surgical procedures (8.0-fold variation for the 95th vs fifth percentile) and lowest among lower abdomen surgical procedures (3.4-fold for the 95th vs fifth percentile). Similar trends were found when individually analyzing anesthesiologist, surgeon, and hospital variation within each of 8 surgical categories (eAppendix 9 and eFigures 3-26 in Supplement 1).

### Adjusted Intraoperative Opioid Administration

ICC estimates were the multilevel methodological approach used to assess the variance attributable to levels of the clinical hierarchical data with patients nested within clinicians, which were nested within hospitals. The proportions of the variance of intraoperative opioid use, or the ICC, from the null model (unadjusted) were all larger than 0.01 (0.77, 0.11, and 0.12 for the patient, clinician, and hospital, respectively), supporting the selection of the generalized linear mixed models method. From here, all reported ICCs are adjusted. Full model metrics for 2 GLMM models can be found in eAppendix 10 and eTable 5 in Supplement 1.

**Figure 2.**
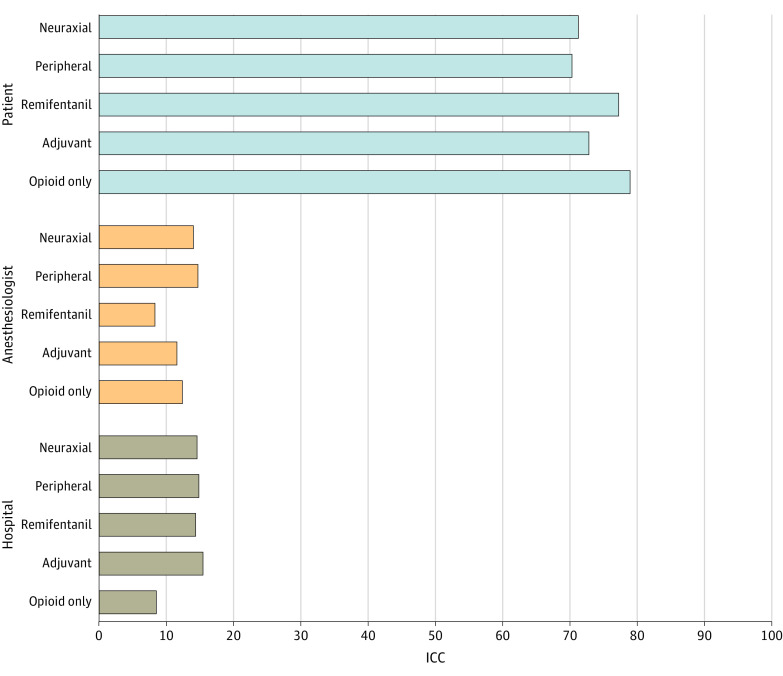
Attributable Variance by Analgesic Technique Adjusted intraclass correlation coefficients (ICCs) of adjusted intraoperative opioid administration are displayed by analgesic category and patient, anesthesiologist, and hospital.

Adjusted ICCs were 0.74, 0.12, and 0.14, for the patient, clinician, and hospital, respectively, in models with anesthesiologists as the clinician. Adjusting for surgical or analgesic category, ICCs ranged from 0.57 to 0.79 for the patient, 0.04 to 0.22 for the anesthesiologist, and 0.09 to 0.26 for the hospital, with the lowest ICC combination 0.21 for anesthesiologist and hospital (0.12 for the anesthesiologist and 0.09 for the hospital for opioid only) (Figure 2; eAppendix 11 and eFigures 27-29 in Supplement 1). When calculated by analgesic category, adjusted ICCs ranged from 0.70 to 0.79 for the patient, 0.08 to 0.15 for the anesthesiologist, and 0.09 to 0.16 for the hospital. When surgeons were used as the clinician, adjusted ICC estimates were 0.61, 0.25, and 0.14 for patient, clinician, and hospital levels, respectively. Surgical category–adjusted ICCs ranged from 0.61 to 0.76 for the patient, 0.02 to 0.11 for the surgeon, and 0.15 to 0.33 for the hospital, while analgesic category–adjusted ICCs ranged from 0.58 to 0.80, 0.07 to 0.30, and 0.09 to 0.15 over the same levels. Across estimations, combined contributions from the clinician and hospital ranged from 0.20 using surgeons and grouping by remifentanil to 0.43 using anesthesiologist and grouping by cardiac surgical procedure.

**Figure 3.**
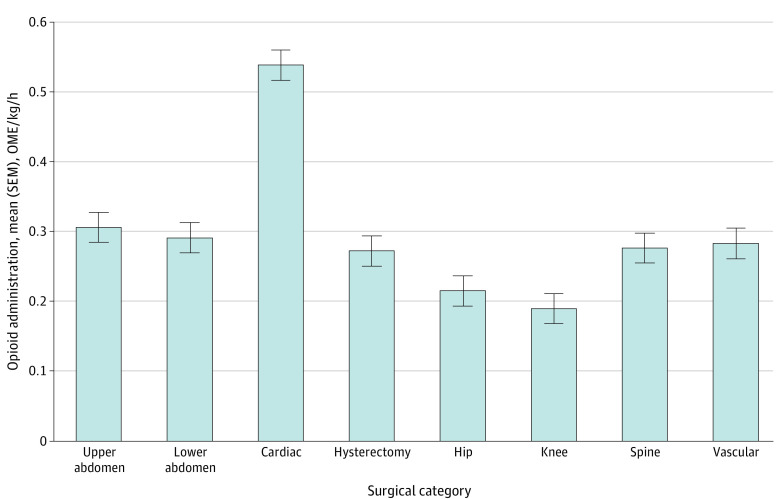
Intraoperative Opioid Administration by Surgical Category Intraoperative opioid administration was measured as the adjusted least square mean–normalized oral morphine equivalency (OME) per kilogram per hour by surgical category. Error bars represent square error means (SEMs).

**Figure 4.**
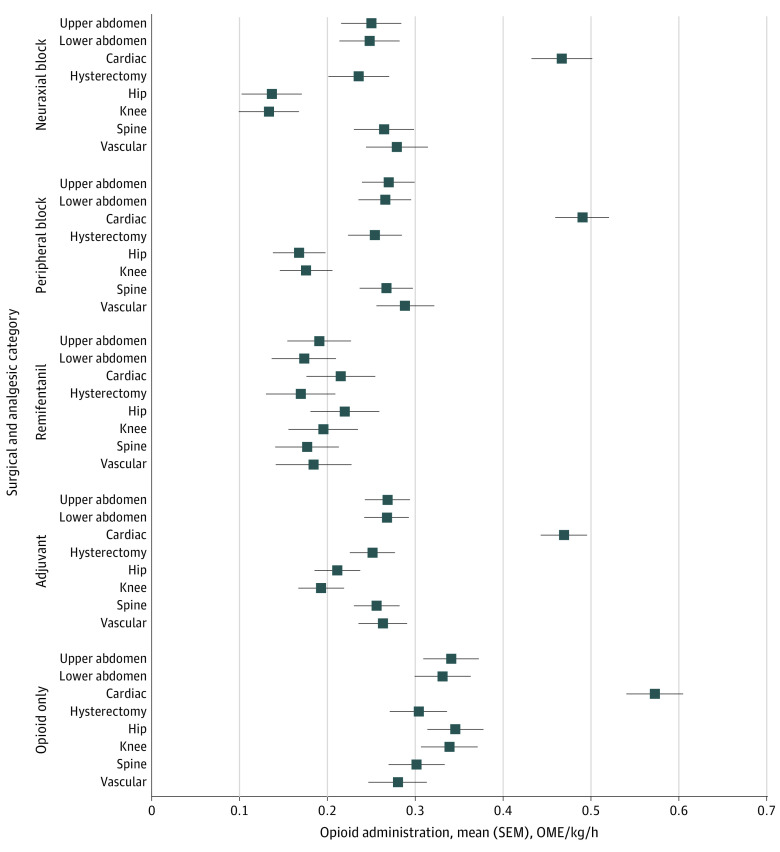
Intraoperative Opioid Administration by Surgical and Analgesic Category Box and whisker plots of adjusted intraoperative opioid administration by surgical and analgesic category are presented. Intraoperative opioid administration was measured as the adjusted least square mean–normalized oral morphine equivalency (OME) per kilogram per hour by type of surgical procedure. Error bars represent square error means (SEMs).

In adjusted analyses, mean (square error mean) opioid administration was highest for cardiac surgical procedures (0.54 [0.56-0.52] OME/kg/h) and lowest for orthopedic knee surgical procedures (0.19 [0.17-0.21] OME/kg/h); similarly, unadjusted analyses found that mean (SD) opioid administration was highest for cardiac surgical procedures (0.36 [0.18] OME/kg/h) and lowest for orthopedic knee surgical procedures (0.17 [0.07] OME/kg/h) (Figure 3). The adjusted mean normalized OME for each analgesic category appears in Figure 4. Intraoperative opioid administration for peripheral or neuraxial techniques had the largest reductions for orthopedic hip surgical procedures (51.6% [95% CI, 51.4%-51.8%] less for peripheral and 60.7% [95% CI ,60.5%-60.9%] less for neuraxial techniques) and orthopedic knee surgical procedures (48.3% [95% CI, 48.0%-48.5%] less for peripheral and 60.9% [95% CI, 60.7%-61.1%] less for neuraxial techniques) compared with opioid only. Mean (SD) differences were smaller for other surgical categories (13.3% [8.8%] less for peripheral and 17.6% [9.9%] less for neuraxial techniques). Cardiac surgical procedures displayed the highest intraoperative opioid administration for each analgesic category except remifentanil. Remifentanil use had lower intraoperative opioid administration across all surgical categories, with a mean (SD) reduction of 44.0% (10.5%), a maximum reduction of 62.5% for cardiac surgical procedures, and a minimum reduction of 27.7% for orthopedic hip surgical procedures. Adjuvant use had a reduction in intraoperative opioid administration across all surgical categories, with a mean (SD) reduction of 22.4% (12.4%).

## Discussion

In this cohort study of more than 1 million surgical procedures using adjusted ICC measurements, we found an important contribution to variation in intraoperative opioid administration at the level of the individual anesthesiologist, surgeon, and hospital across multiple surgical types and analgesic techniques, with clinician and hospitals combining to 0.20 or greater in all analyses. These results may be clinically significant given that this design resembles the natural structure of health care practice, in which patients are treated by clinicians who work within hospitals. Furthermore, results from this 3-level hierarchical approach are statistically relevant given that statistical practices recognize clustering contributions for ICCs as low as 0.01 to be statistically significant.^27^ ICC estimates are common in health research and allow proportioning of variation in an outcome.^29^ Studies that fail to consider contributions of each hierarchical level are likely to result in incorrect conclusions, and when nonindependent data in a nested structure are incorrectly assumed to be independent, statistically significant results may occur even when there is no association with treatment outcomes. Our findings suggest that anywhere from 20% to 43% of the variation in intraoperative opioid administration may be not accounted for if analyses ignore clinician and hospital factors and rely on only patient characteristics.

We found a large variation of opioid administration between individual hospitals and clinicians, showing unadjusted OME variation overall of 34.3-fold among anesthesiologists, 23.0-fold among surgeons, and 5.0-fold among hospitals and a variation using 95th vs fifth percentiles of 3.3-fold, 4.3-fold, and 2.2-fold for anesthesiologists, surgeons, and hospitals, respectively. Cardiac surgical procedures displayed the highest OME administration across analyses, which may be expected given that anesthesia for these surgical procedures has historically relied on methods of high opioid administration (including high-dose sufentanil infusions and bolus doses >1500 OME) not in widespread use for other surgical procedures, warranting further investigation. Orthopedic knee and hip surgical procedures had among the lowest administration levels, which may reflect widespread use of ERAS protocols for orthopedic surgical procedures. We discovered a consistent pattern of variation across analyses of all surgical categories. When plotting intraoperative opioid administration, there was a clear pattern of increased starting and high ending slopes, with a moderate linear increase in between, evident in the large reduction from overall to ninety-fifth vs fifth percentile fold comparisons. This pattern persisted when considering clinicians and hospitals, suggesting that most clinicians (surgeons and anesthesiologists) and hospitals used opioids in a broad dosing window, while outliers existed on both extremes.

Supplemental analgesic techniques are often used to minimize patient discomfort and limit opioid administration throughout the perioperative period. In our study, we found that all nonopioid analgesic techniques were associated with decreased intraoperative opioid administration relative to opioid-only methods, further supporting incorporation of multimodal techniques in opioid-sparing efforts. Adjuvant use was more variable, which is not surprising given that nonopioid adjuvants span a wide array of drug classes and pain control potential. Furthermore, the use of neuraxial and peripheral nerve blocks was associated with the greatest OME reduction in orthopedic knee and hip procedures. This, again, may be due to existing efforts in opioid-reduction strategies and ERAS protocols in these 2 surgical types or characteristics of the procedures and outcomes associated with these analgesic techniques. Remifentanil and adjuvant use were independently associated with decreased OME levels across all surgical procedure types. Remifentanil is an easily titratable opioid medication with a nonexistent context-sensitive half-time. Its operative use likely supplants the use of other, longer-acting intraoperative opioids, which may lower the total intraoperative opioid administered. Simultaneously, its use may be associated with worse postoperative pain pathways given that prior studies found that intraoperative remifentanil use was associated with increased postoperative pain scores and subsequent opioid administration owing to acute opioid tolerance and opioid-induced hyperalgesia.^30,31^

The opioid overdose epidemic in the US^32^ has prompted reevaluation of how these medications are clinically used.^33^ This is especially important in the perioperative period, in which opioid use before a surgical procedure has as high a rate as 1 in 4 patients undergoing surgical procedures^34^ and most patients undergoing surgical procedures who are opioid naive will be newly exposed to these medications intraoperatively or prescribed them after discharge from a surgical procedure.^2^ Several studies suggest that prescription opioid use after discharge is a risk factor associated with chronic opioid use,^35,36,37,38,39^ specifically after major elective operations.^40^ An unresolved question is the extent to which intraoperative opioid administration contributes to worse opioid-related outcomes after surgical procedures, highlighting the need for further investigations.^7^ Existing perioperative analgesic plans can be complicated, with several overlapping contributors to intraoperative opioid administration, such as patient demographics, opioid medication route and dose considerations, adjuvant medication use, and analgesic techniques, including neuraxial analgesia and peripheral nerve blocks. Focused studies investigating the effect of intraoperative opioid administration lack broad understanding of the sources of variation. As a result, it is unclear based on current available evidence that opioids administered intraoperatively are associated with surgical outcomes or increased risk for aberrant opioid use postoperatively.^41,42,43,44,45^ Recent studies suggested the importance of understanding qualitative and quantitative aspects of intraoperative opioid administrations for postoperative pain and opioid consumption.^46,47^ Characterizing heterogeneity across clinicians and practices may provide valuable insight to elucidate patterns, improve guidelines, and influence policy change to promote better stewardship of opioids. Variation in intraoperative opioid administration remains an important area of investigation, without which efforts to improve patient recovery, such as ERAS, will be isolated.

### Limitations

There exist several important limitations to this study. First, this study focused on adult patients who were opioid naive and undergoing a surgical procedure from 1 of 8 surgical categories at 1 of 46 hospitals. While practice settings spanned multiple states and all regions of the US, results may not be representative of other surgical procedures, patient populations, or practice locations. Second, the classification of analgesic techniques may aggregate important characteristics that deserve more granular analysis, such as inclusion of both epidural and spinal approaches within the neuraxial technique. Third, intraoperative opioid administration calculations depend on proper documentation of the medication dose, route, and timing within the EHR. Fourth, the timing of medication dosing and analgesic techniques, which may influence the intraoperative dose of opioids, were not available and were not included in this analysis. Fifth, while NPI data permitted following anesthesiologists across hospitals, this linkage was not possible for surgeons, who were considered as unique clinicians in this study. Sixth, normalized opioid equivalency calculations are challenging given that potencies vary across routes and medications, equipotent medications may have dramatically different durations of action, and there exists wide variation in accepted equianalgesic ratios. Pharmacokinetics (eg, short vs long acting) should be considered. For example, a single dose of fentanyl has a short-lasting effect despite a high OME dose but would be different than an equivalent OME dose of a long-acting opioids, such as hydromorphone or morphine. Further analysis is warranted.

## Conclusion

In this cohort study of 1 011 268 surgical procedures at 46 hospitals across the US investigating surgical opioid administration variation, important contributions were found at clinician and hospital levels, underscoring the need to consider both factors in addition to patient and procedural characteristics. Characterizing heterogeneity across clinicians and practices may provide valuable insight to help identify patterns, improve guidelines, and influence policy change to promote better stewardship of opioids. The observed variation in this study may be significant in subsequent phases of patient care, such as the postanesthesia care unit, floor, intensive care unit, and after discharge. With these findings, we may be able to investigate the creation of personalized opioid-dosing plans based on patient and operative characteristics, improve and expand effective ERAS protocols, and study potential associations between surgical opioid variation and adverse outcomes in the postanesthesia care unit and beyond.

## Notes

Supplement 1. **eAppendix 1.** Surgical Grouping by Current Procedural Terminology Code **eAppendix 2.** Analytical Dataset Characteristics **eAppendix 3.** *ICD-9* and *ICD-10* Codes Used to Determine Use and Abuse History **eAppendix 4.** Preoperative Opioid Medications List **eAppendix 5.** Analgesic Technique Components **eAppendix 6.** Analgesic Adjuvant Medication List **eAppendix 7.** Oral Morphine Equivalency Conversions **eAppendix 8.** Individual Hospitals Ranked by Intraoperative Opioid Administration **eAppendix 9.** Anesthesiologist, Surgeon, and Hospital Variation by Surgical Category **eAppendix 10.** Full Model Results for Generalized Linear Mixed Model With Random Intercept **eAppendix 11.** Adjusted Intraclass Correlation Coefficients by Surgical Group or Analgesic Category **eTable 1.** Surgical Grouping by *Current Procedural Terminology* Code **eTable 2.** Characteristics of Full Dataset **eTable 3.** *ICD-9* and *ICD-10* Codes Used to Determine Use and Abuse History **eTable 4.** Oral Morphine Equivalency Conversions **eTable 5.** Full Model Results for Generalized Linear Mixed Model With Random Intercept **eFigure 1.** Study Flowchart **eFigure 2.** Individual Hospitals Ranked by Intraoperative Opioid Administration **eFigure 3.** Intraoperative Opioid Administration by Anesthesiologist for Cardiac Surgeries **eFigure 4.** Intraoperative Opioid Administration by Anesthesiologist for Hip Surgeries **eFigure 5.** Intraoperative Opioid Administration by Anesthesiologist for Hysterectomy Surgeries **eFigure 6.** Intraoperative Opioid Administration by Anesthesiologist for Knee Surgeries **eFigure 7.** Intraoperative Opioid Administration by Anesthesiologist for Lower Abdominal Surgeries **eFigure 8.** Intraoperative Opioid Administration by Anesthesiologist for Vascular Surgeries **eFigure 9.** Intraoperative Opioid Administration by Anesthesiologist for Spine Surgeries **eFigure 10.** Intraoperative Opioid Administration by Anesthesiologist for Upper Abdominal Surgeries **eFigure 11.** Intraoperative Opioid Administration by Surgeon for Cardiac Surgeries **eFigure 12.** Intraoperative Opioid Administration by Surgeon for Hip Surgeries **eFigure 13.** Intraoperative Opioid Administration by Surgeon for Hysterectomy Surgeries **eFigure 14.** Intraoperative Opioid Administration by Surgeon for Knee Surgeries **eFigure 15.** Intraoperative Opioid Administration by Surgeon for Lower Abdominal Surgeries **eFigure 16.** Intraoperative Opioid Administration by Surgeon for Vascular Surgeries **eFigure 17.** Intraoperative Opioid Administration by Surgeon for Spine Surgeries **eFigure 18.** Intraoperative Opioid Administration by Surgeon for Upper Abdominal Surgeries **eFigure 19.** Intraoperative Opioid Administration by Hospital for Cardiac Surgeries **eFigure 20.** Intraoperative Opioid Administration by Hospital for Hip Surgeries **eFigure 21.** Intraoperative Opioid Administration by Hospital for Hysterectomy Surgeries **eFigure 22.** Intraoperative Opioid Administration by Hospital for Knee Surgeries **eFigure 23.** Intraoperative Opioid Administration by Hospital for Lower Abdominal Surgeries **eFigure 24.** Intraoperative Opioid Administration by Hospital for Vascular Surgeries **eFigure 25.** Intraoperative Opioid Administration by Hospital for Spine Surgeries **eFigure 26.** Intraoperative Opioid Administration by Hospital for Upper Abdominal Surgeries **eFigure 27.** Intraclass Correlation Coefficients of Adjusted Intraoperative Opioid Administration by Surgical Category and Patient, Anesthesiologist, and Hospital **eFigure 28.** Intraclass Correlation Coefficients of Adjusted Intraoperative Opioid Administration by Surgical Category and Patient, Surgeon, and Hospital **eFigure 29.** Intraclass Correlation Coefficients of Adjusted Intraoperative Opioid Administration by Analgesic Category and Patient, Surgeon, and Hospital

Supplement 2.
Data Sharing Statement
