# Supplementary material for: Variation in Intraoperative Opioid Administration by Patient, Clinician, and Hospital Contribution
Source: JAMA Netw Open. 2024 Jan 16;7(1):e2351689. doi: 10.1001/jamanetworkopen.2023.51689 (PMC10792468; doi:10.1001/jamanetworkopen.2023.51689)
Supplement: Supplement 1. — eAppendix 1. Surgical Grouping by Current Procedural Terminology Code eAppendix 2. Analytical Dataset Characteristics eAppendix 3. ICD-9 and ICD-10 Codes Used to Determine Use and Abuse History eAppendix 4. Preoperative Opioid Medications List eAppendix 5. Analgesic Technique Components eAppendix 6. Analgesic Adjuvant Medication List eAppendix 7. Oral Morphine Equivalency Conversions eAppendix 8. Individual Hospitals Ranked by Intraoperative Opioid Administration eAppendix 9. Anesthesiologist, Surgeon, and Hospital Variation by Surgical Category eAppendix 10. Full Model Results for Generalized Linear Mixed Model With Random Intercept eAppendix 11. Adjusted Intraclass Correlation Coefficients by Surgical Group or Analgesic Category eTable 1. Surgical Grouping by Current Procedural Terminology Code eTable 2. Characteristics of Full Dataset eTable 3. ICD-9 and ICD-10 Codes Used to Determine Use and Abuse History eTable 4. Oral Morphine Equivalency Conversions eTable 5. Full Model Results for Generalized Linear Mixed Model With Random Intercept eFigure 1. Study Flowchart eFigure 2. Individual Hospitals Ranked by Intraoperative Opioid Administration eFigure 3. Intraoperative Opioid Administration by Anesthesiologist for Cardiac Surgeries eFigure 4. Intraoperative Opioid Administration by Anesthesiologist for Hip Surgeries eFigure 5. Intraoperative Opioid Administration by Anesthesiologist for Hysterectomy Surgeries eFigure 6. Intraoperative Opioid Administration by Anesthesiologist for Knee Surgeries eFigure 7. Intraoperative Opioid Administration by Anesthesiologist for Lower Abdominal Surgeries eFigure 8. Intraoperative Opioid Administration by Anesthesiologist for Vascular Surgeries eFigure 9. Intraoperative Opioid Administration by Anesthesiologist for Spine Surgeries eFigure 10. Intraoperative Opioid Administration by Anesthesiologist for Upper Abdominal Surgeries eFigure 11. Intraoperative Opioid Administration by Surgeon for Cardiac Surgeries eFigure 12. Intraoper [file jamanetwopen-e2351689-s001.pdf]

Supplemental Online Content

Burns ML, Hilliard P, Vandervest J, et al. Variation in intraoperative opioid administration by patient, clinician, and hospital contribution. *JAMA Netw Open*. 2024;7(1):e2351689. doi:10.1001/jamanetworkopen.2023.51689

eAppendix 1. Surgical Grouping by Current Procedural Terminology Code

eAppendix 2. Analytical Dataset Characteristics

eAppendix 3. *ICD-9* and *ICD-10* Codes Used to Determine Use and Abuse History

eAppendix 4. Preoperative Opioid Medications List

eAppendix 5. Analgesic Technique Components

eAppendix 6. Analgesic Adjuvant Medication List

eAppendix 7. Oral Morphine Equivalency Conversions

eAppendix 8. Individual Hospitals Ranked by Intraoperative Opioid Administration

eAppendix 9. Anesthesiologist, Surgeon, and Hospital Variation by Surgical Category

eAppendix 10. Full Model Results for Generalized Linear Mixed Model With Random Intercept

eAppendix 11. Adjusted Intraclass Correlation Coefficients by Surgical Group or Analgesic Category

eTable 1. Surgical Grouping by *Current Procedural Terminology* Code

eTable 2. Characteristics of Full Dataset

eTable 3. *ICD-9* and *ICD-10* Codes Used to Determine Use and Abuse History

eTable 4. Oral Morphine Equivalency Conversions

eTable 5. Full Model Results for Generalized Linear Mixed Model With Random Intercept

eFigure 1. Study Flowchart

eFigure 2. Individual Hospitals Ranked by Intraoperative Opioid Administration

eFigure 3. Intraoperative Opioid Administration by Anesthesiologist for Cardiac Surgeries

eFigure 4. Intraoperative Opioid Administration by Anesthesiologist for Hip Surgeries

eFigure 5. Intraoperative Opioid Administration by Anesthesiologist for Hysterectomy Surgeries

eFigure 6. Intraoperative Opioid Administration by Anesthesiologist for Knee Surgeries

eFigure 7. Intraoperative Opioid Administration by Anesthesiologist for Lower Abdominal Surgeries

eFigure 8. Intraoperative Opioid Administration by Anesthesiologist for Vascular Surgeries

eFigure 9. Intraoperative Opioid Administration by Anesthesiologist for Spine Surgeries

© 2024 Burns ML et al. *JAMA Network Open.*

eFigure 10. Intraoperative Opioid Administration by Anesthesiologist for Upper Abdominal Surgeries

eFigure 11. Intraoperative Opioid Administration by Surgeon for Cardiac Surgeries

eFigure 12. Intraoperative Opioid Administration by Surgeon for Hip Surgeries

eFigure 13. Intraoperative Opioid Administration by Surgeon for Hysterectomy Surgeries

eFigure 14. Intraoperative Opioid Administration by Surgeon for Knee Surgeries

eFigure 15. Intraoperative Opioid Administration by Surgeon for Lower Abdominal Surgeries

eFigure 16. Intraoperative Opioid Administration by Surgeon for Vascular Surgeries

eFigure 17. Intraoperative Opioid Administration by Surgeon for Spine Surgeries

eFigure 18. Intraoperative Opioid Administration by Surgeon for Upper Abdominal Surgeries

eFigure 19. Intraoperative Opioid Administration by Hospital for Cardiac Surgeries

eFigure 20. Intraoperative Opioid Administration by Hospital for Hip Surgeries

eFigure 21. Intraoperative Opioid Administration by Hospital for Hysterectomy Surgeries

eFigure 22. Intraoperative Opioid Administration by Hospital for Knee Surgeries

eFigure 23. Intraoperative Opioid Administration by Hospital for Lower Abdominal Surgeries

eFigure 24. Intraoperative Opioid Administration by Hospital for Vascular Surgeries

eFigure 25. Intraoperative Opioid Administration by Hospital for Spine Surgeries

eFigure 26. Intraoperative Opioid Administration by Hospital for Upper Abdominal Surgeries

eFigure 27. Intraclass Correlation Coefficients of Adjusted Intraoperative Opioid Administration by Surgical Category and Patient, Anesthesiologist, and Hospital

eFigure 28. Intraclass Correlation Coefficients of Adjusted Intraoperative Opioid Administration by Surgical Category and Patient, Surgeon, and Hospital

eFigure 29. Intraclass Correlation Coefficients of Adjusted Intraoperative Opioid Administration by Analgesic Category and Patient, Surgeon, and Hospital

This supplemental material has been provided by the authors to give readers additional information about their work.

© 2024 Burns ML et al. *JAMA Network Open.*

Supplemental Material 1
Surgical Grouping by Current Procedural Terminology (CPT)

Primary Anesthesia CPT	Description	Percentage of Total Cases (%)
**Lower Abdomen**		**24.3%**
00840	Anesthesia for intraperitoneal procedures in lower abdomen including laparoscopy; not otherwise specified	23.9%
00844	Anesthesia for intraperitoneal procedures in lower abdomen including laparoscopy; abdominoperineal resection	0.3%
00848	Anesthesia for intraperitoneal procedures in lower abdomen including laparoscopy; pelvic exenteration	0.1%
**Upper Abdomen**		**32.0%**
00790	Anesthesia for intraperitoneal procedures in upper abdomen including laparoscopy; not otherwise specified	27.6%
00797	Anesthesia for intraperitoneal procedures in upper abdomen including laparoscopy; gastric restrictive procedure for morbid obesity	2.9%
00792	Anesthesia for intraperitoneal procedures in upper abdomen including laparoscopy; partial hepatectomy or management of liver hemorrhage (excluding liver biopsy)	0.8%
00794	Anesthesia for intraperitoneal procedures in upper abdomen including laparoscopy; pancreatectomy, partial or total (eg, Whipple procedure)	0.7%
**Cardiac**		**9.0%**
00562	Anesthesia for procedures on heart, pericardial sac, and great vessels of chest; with pump oxygenator, age 1 year or older, for all non-coronary bypass procedures (eg, valve procedures) or for re-operation for coronary bypass more than 1 month after original operation	6.1%
00567	Anesthesia for direct coronary artery bypass grafting; with pump oxygenator	1.7%
00563	Anesthesia for procedures on heart, pericardial sac, and great vessels of chest; with pump oxygenator with	0.6%

© 2024 Burns ML et al. *JAMA Network Open.*

	hypothermic circulatory arrest	
00580	Anesthesia for heart transplant or heart/lung transplant	0.5%
00566	Anesthesia for direct coronary artery bypass grafting; without pump oxygenator	0.1%
**Hysterectomy**		**1.6%**
00944	Anesthesia for vaginal procedures (including biopsy of labia, vagina, cervix or endometrium); vaginal hysterectomy	1.0%
00846	Anesthesia for intraperitoneal procedures in lower abdomen including laparoscopy; radical hysterectomy	0.6%
**Orthopedic Hip**		**6.1%**
01214	Anesthesia for open procedures involving hip joint; total hip arthroplasty	6.1%
**Orthopedic Knee**		**8.0%**
01402	Anesthesia for open or surgical arthroscopic procedures on knee joint; total knee arthroplasty	8.0%
**Orthopedic Spine**		**18.8%**
00670	Anesthesia for extensive spine and spinal cord procedures (eg, spinal instrumentation or vascular procedures)	9.7%
00630	Anesthesia for procedures in lumbar region; not otherwise specified	6.3%
00600	Anesthesia for procedures on cervical spine and cord; not otherwise specified	1.7%
00620	Anesthesia for procedures on thoracic spine and cord; not otherwise specified	0.6%
00625	Anesthesia for procedures on the thoracic spine and cord, via an anterior transthoracic approach; not utilizing 1 lung ventilation	<0.1%
00626	Anesthesia for procedures on the thoracic spine and cord, via an anterior transthoracic approach; utilizing 1 lung ventilation	<0.1%
00632	Anesthesia for procedures in lumbar region; lumbar	<0.1%

© 2024 Burns ML et al. *JAMA Network Open.*

	sympathectomy	
00622	Anesthesia for procedures on thoracic spine and cord; thoracolumbar sympathectomy	<0.1%
**Vascular**		**0.4%**
00880	Anesthesia for procedures on major lower abdominal vessels; not otherwise specified	0.4%
00882	Anesthesia for procedures on major lower abdominal vessels; inferior vena cava ligation	<0.1%

eTable 1: Surgical Grouping by Current Procedural Terminology (CPT) Code.

Surgeries were grouped by Current Procedural Terminology (CPT) codes into eight categories: lower abdomen, upper abdomen, cardiac, hysterectomy, orthopedic hip, orthopedic knee, orthopedic spine, and vascular). Percentage of the total cases is shown by category as well as individual CPT code.

© 2024 Burns ML et al. *JAMA Network Open.*

Supplemental Material 2
Analytical Dataset Characteristics

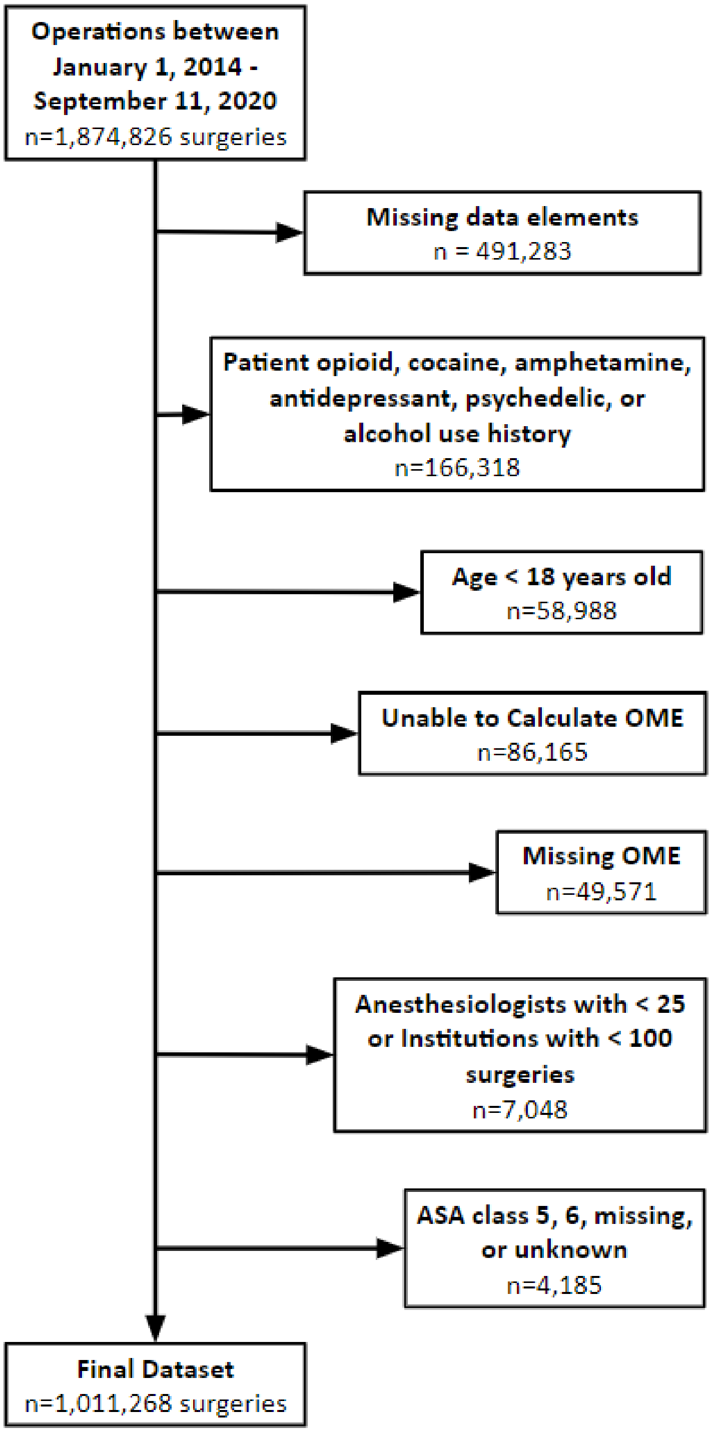

eFigure 1: Study flowchart.

© 2024 Burns ML et al. *JAMA Network Open.*

Exclusion criteria were applied to the overall starting population to result in the Final Dataset (n = 1,012,592). Missing data elements consist of missing patient weight, CPT codes, or case duration. Unable to calculate OME results from inappropriate medication dose and unit of measure data. OME = Oral Morphine Equivalency.

Measure	N	Mean	Stdev	25th	75th
OME Normalized	1011268	0.3	0.2	0.2	0.4
Age (years)	1011268	55.9	16.2	44.0	68.0
Body Mass Index	986856	30.4	7.7	25.0	34.4
Duration (min)	1011268	168.8	127.0	87.0	218.0

Measure	Level	N	%
Gender	Female	604057	59.7
	Male	407069	40.3
	Unknown	142	0.0
Race	NH Black	115492	11.4
	NH White	764365	75.6
	Other	131411	13.0
Categorical Age	18-40	201976	20.0
	41-50	155373	15.4
	51-60	209957	20.8
	61-70	243564	24.1
	71+	200398	19.8
BMI Classification	Class I Obesity	212294	21.0
	Class II Obesity	119767	11.8
	Class III Obesity	107546	10.6
	Normal or Underweight	274442	27.1
	Overweight	297219	29.4
ASA Class	ASA Class 1	53850	5.3
	ASA Class 2	408223	40.4
	ASA Class 3	442949	43.9
	ASA Class 4	104776	10.4
Emergency Status	No	910358	90.0
	Yes	75356	7.5
	Unmapped	24094	2.4
	Missing	1460	0.1
Weekend	No	962823	95.2

© 2024 Burns ML et al. *JAMA Network Open.*

	Yes	48445	4.8
Holiday	No	1007712	99.6
	Yes	3556	0.4
Surgical Group	Abdomen: Upper	303883	30.0
	Abdomen: Lower	256715	25.4
	Orthopedics: Spine	187448	18.5
	Orthopedics: Knee	89890	8.9
	Cardiac	80118	7.9
	Orthopedics: Hip	65932	6.5
	Hysterectomy	23087	2.3
	Vascular	4195	0.4
**Elixhauser Comorbidity**	**Level**	**N**	**%**
Aids HIV	No	1009395	99.8
	Yes	1873	0.2
Alcohol Abuse	No	1011268	100.0
	Yes	0	0.0
Blood Loss Anemia	No	997651	98.7
	Yes	13617	1.3
Cardiac Arrhythmias	No	846494	83.7
	Yes	164773	16.3
Chronic Pulmonary Disease	No	859080	85.0
	Yes	152187	15.0
Coagulopathy	No	946250	93.6
	Yes	65017	6.4
Congestive Heart Failure	No	935682	92.5
	Yes	75569	7.5
Deficiency Anemia	No	983218	97.2
	Yes	28049	2.8
Depression	No	877088	86.7
	Yes	134179	13.3
Diabetes Complicated	No	977955	96.7
	Yes	33296	3.3
Diabetes Uncomplicated	No	891777	88.2
	Yes	119474	11.8
Drug Abuse	No	990378	97.9
	Yes	20889	2.1
Fluid Electrolyte Disorder	No	849939	84.0
	Yes	161328	16.0
Hypertension Complicated	No	918179	90.8

© 2024 Burns ML et al. *JAMA Network Open.*

	Yes	93088	9.2
Hypertension Uncomplicated	No	622486	61.6
	Yes	388781	38.4
Hypothyroidism	No	902181	89.2
	Yes	109086	10.8
Liver Disease	No	961268	95.1
	Yes	49999	4.9
Lymphoma	No	1004492	99.3
	Yes	6775	0.7
Metastatic Cancer	No	951262	94.1
	Yes	60005	5.9
Obesity	No	781784	77.3
	Yes	229483	22.7
Other Neurological Disorder	No	970861	96.0
	Yes	40406	4.0
Paralysis	No	999389	98.8
	Yes	11878	1.2
Peptic Ulcer Disease	No	1002860	99.2
	Yes	8407	0.8
Peripheral Vascular Disease	No	945340	93.5
	Yes	65927	6.5
Psychoses	No	1006217	99.5
	Yes	5050	0.5
Pulmonary Circulation Disease	No	977744	96.7
	Yes	33523	3.3
Renal Failure	No	932105	92.2
	Yes	79162	7.8
Rheumatoid Arthritis	No	979367	96.8
	Yes	31900	3.2
Solid Tumor w/o Metastasis	No	890596	88.1
	Yes	120671	11.9
Valvular Disease	No	928276	91.8
	Yes	82991	8.2
Weight Loss	No	949706	93.9
	Yes	61561	6.1

eTable 2: Characteristics of the full dataset. NH=non-Hispanic.

© 2024 Burns ML et al. *JAMA Network Open.*

1. Elixhauser A, Steiner C, Harris DR, Coffey RM. Comorbidity measures for use with administrative data. Med Care. 1998 Jan;36(1):8-27. doi: 10.1097/00005650-199801000-00004. PMID: 9431328.

© 2024 Burns ML et al. *JAMA Network Open.*

Supplemental Material 3
International Classification of Diseases (ICD) Codes Used to Determine Use/Abuse History

ICD-9 and ICD-10 Codes	Description
**Opioid History**	
F11.1%<br>30550-30553%	opioid abuse
F11.2%<br>30403%	opioid dependence
T40.0-T40.4%<br>9650%	overdose or poisoning by heroin, methadone, other opiates and related narcotics
3047%<br>F19.2%	opioid in combination with another drug dependence
F11.9%	opioid use
**Cocaine and Amphetamine History**	
F15.1%<br>30570-30573%	amphetamine abuse
F15.2%<br>30440-30443%	amphetamine dependence
T43.62%<br>969.72	poisoning by amphetamines
F15.2%<br>3044%	amphetamine dependence
F14.1%<br>30560-30563%	cocaine abuse
F14.9%	cocaine dependence
T40.5%<br>97081%	cocaine poisoning, adverse effects
**Antidepressant and Psychoactive History**	
305.8%	antidepressant abuse
T43.0%-T43.2%<br>969.0%	antidepressant poisoning

© 2024 Burns ML et al. *JAMA Network Open.*

F19.1%	other psychoactive abuse, substance abuse
F19.2%	other psychoactive substance dependence
**Alcohol Abuse**	
980.%<br>T51.%	Toxic effect of (an alcohol)
265.2<br>E52	Pellagra (Niacin deficiency)
291.[1-35-9].%<br>303.[09]%<br>305.0%<br>F10	Alcohol abuse (withdrawal, mental changes), Acute alcohol intoxication, Non-dependent Alcohol abuse
357.5%<br>G62.1%	Alcoholic polyneuropathy
425.5%<br>I42.6%	Alcoholic cardiomyopathy
535.3%<br>K29.2%	Alcoholic Gastritis
571.[0-3]%<br>K70.[039]%	Alcoholic fatty liver, hepatitis, cirrhosis
V11.3%<br>Z72.1%	Problem w/ alcohol use
Z50.2%<br>Z71.4%	Alcohol Abuse Counseling / Rehab

eTable 3: International Classification of Diseases (ICD) Codes Used to Determine Use/Abuse History.

ICD 9 and 10 codes used to determine use/abuse history from opioids, cocaine/amphetamines, antidepressants/psychoactives, and alcohol.

% denotes wildcard, meaning any type and any number of alphanumeric characters may replace the % and be accepted.

© 2024 Burns ML et al. *JAMA Network Open.*

Supplemental Material 4
Preoperative Opioid Medications List

Abstral, Acetaco, Acetatab, Actiq, Alfentanil, Allay, Alor 5/500, Amacodone, Anexsia, Anolor, Arymo, Avinza, Azdone, B & O Supprettes, B-A-C #3, Balacet, Bancap, Belbuca, Bexophene, Bunavail, Buprenorphin, Buprenorphine, But/Apap/Caff/Cod, Butorphanol, Butrans, Cefaclor, Ceta Plus, Coastaldyne, Cocet, Codaphen, Codeine, Co-Gesic, Combunox, Conzip, Cotanal-65, Damason-P, Darvocet, Darvon, Dazidox, Demerol, Dhc Plus, Dihydrocodei, Dihydrocodeine, Dilaudid, Diskets Dispersible, Dolacet, Dolagesic, Dolene, Dolfen, Dolo-Pap, Dolophine, Dolorex, Doraphen, Doxapap, Doxaphene, D-Rex 65, Duocet, Duradyne, Duragesic, Dvorah, E-Lor, Embeda, Emcodeine, Empracet, Endocet, Endocodone, Endodan, Eth-Oxydose, Exalgo, Ez Iii, Febridyne, Fentanyl, Fentora, Fusepaq Synapryn, G-3, Gesic 5, Hy-5, Hycet, Hycogesic, Hycomed, Hyco-Pap, Hycotab, Hydrocet, Hydrocod Bit & Acet, Hydrocodone, Hydrogesic, Hydromorphon, Hydromorphone, Hydrostat, Hy-Phen, Hysingla, Ibudone, Idenal, Ionsys, Isobutal, Kadian, Lazanda, Levo-Dromoran, Levomethadyl, Levorphanol, Liquicet, Lorcet, Lortab, Magnacet, Margesic, Margesic-H, Maxidone, Medipain 5, Megagesic, Megamor, Meperedine, Meperidine, Meperitab, Methadone, Methadose, M-Gesic, Morphabond, Morphine, Ms Contin, Mscontin, Msir, Nalbuphine, Nalocet, Naloxone, Naltrexone, Narvox, Norcet, Norco, Nucynta, Numorphan, Oncet, Onsolis, Opana, Opium, Oramorph, Orlaam, Oxaydo, Oxecta, Oxy Ir, Oxycet, Oxycodone, Oxycontin, Oxyfast, Oxymorphone, Palladone, Panacet, Panasal, Pancet, Panlor, Pc-Cap, Pentazocine, Percocet, Percodan, Percodan-Demi, Percolone, Perloxx, Polygesic, Pp-Cap, Primalev, Primlev, Pro Pox, Probuphine, Procet, Pronap-100, Propacet, Propain, Propoxacet, Propoxy, Propoxy Naps/Apap, Propoxycon, Propoxyphene, Proval, Pyregesic-C, Regal-Dc, Remifentanil, Reprexain, Rescudose, Ro-Codone, Rogesic, Roxanol, Roxicet, Roxicodone, Roxiprin, Roxybond, Rybix Odt, Ryzolt, Senefen, Sk-65, Stadol, Stagesic, Sublocade, Suboxone, Subsys, Subutex, Sufentanil, Synalgos-Dc, Talacen, Talwin Compound, Talwin Nx, Tapentadol, T-Gesic, Tramadol, Trezix, Tylox, Ugesic, Ultracet, Ultram,

© 2024 Burns ML et al. *JAMA Network Open.*

Vanacet, Vapocet, Vendone, Verdrocet, Vicodin, Vicoprofen, Vopac, Wygesic, Xartemis, Xodol, Xolox, Xtampza, Xylon, Zamicet, Zerlor, Zohydro, Zolvit, Zubsolv, Zydone

This list of medications is adapted from: CDC File of National Drug Codes for Selected Benzodiazepines, Muscle Relaxants, Stimulants, Opioid analgesics, and Linked Oral Morphine Milligram Equivalent Conversion Factors for Opioids, 2019 Version. Atlanta, GA: Centers for Disease Control and Prevention; 2020. Available upon request at https://www.cdc.gov/opioids/data-resources/index.html

© 2024 Burns ML et al. *JAMA Network Open.*

Supplemental Material 5
Analgesic Technique Components

One surgery may have more than one analgesic technique (e.g., neuraxial analgesia and remifentanil), except for 'Opioid Only' which was absent from all other techniques. Remifentanil has its own category due to its common intraoperative use and unique pharmacokinetics including rapid plasma metabolization and short context-sensitive half-life. Neuraxial and peripheral categories captured specific nerve blocks identified through note concepts, key word searches, and administered medications. The remifentanil category was defined as the use of the medication remifentanil between anesthesia start and anesthesia end times. The adjuvant category was defined as adjuvant medication use between one hour before anesthesia start to anesthesia end times and given by intravenous, oral, or enteric tube route of delivery (Supplemental Material 6).

Neuraxial and peripheral nerve block phenotypes were created by the coordinating center and return the values detailed below. These phenotypes search note concepts, key words within clinical notes, and medications to define neuraxial and peripheral blocks for each surgery.

Neuraxial Nerve Block: Yes = any of the following as defined by the phenotype (Version 1): https://phenotypes.mpog.org/Anesthesia%20Technique:%20Neuraxial

1. Neuraxial - Unknown Type: The surgery used a Neuraxial anesthesia technique, but the algorithm was unable to determine the type based on the documentation.
2. Neuraxial - Multiple Types Listed: The surgery used/attempted neuraxial blocks, more than one specific type identified.
3. The surgery used/attempted any of the following blocks: Combined Spinal Epidural (CSE), Epidural, Spinal, Caudal

© 2024 Burns ML et al. *JAMA Network Open.*

Peripheral Nerve Block: Yes = any of the following as defined by the phenotype (Version 2): https://phenotypes.mpog.org/Anesthesia%20Technique:%20Peripheral%20Nerve%20Block

1. Block, Unknown Type: The surgery used/attempted a peripheral block, specific type could not be identified.
2. Block, Multiple Types Listed: The surgery used/attempted peripheral blocks, more than one specific type identified.
3. The surgery used/attempted any of the following blocks: Cervical Plexus, Adductor Canal, Supraclavicular, Interscalene, Infraclavicular, Axillary, Femoral Nerve, Popliteal, Sciatic, Ankle, Bier, Lumbar Plexus, Retrobulbar, Superior Laryngeal, Saphenous Nerve, Inguinal, Transversus Abdominis Plane (TAP), Perineural Block, Eye Block - unspecified, Quadratus Lumborum, Fascia Iliac

© 2024 Burns ML et al. *JAMA Network Open.*

Supplemental Material 6
Analgesic Adjuvant Medication List

Adjuvant medications were considered from the following list:

Acetaminophen, Aspirin, Celecoxib, Diclofenac, Dexamethasone, Dexmedetomidine, Gabapentin, Ibuprofen, Ketamine, Ketorolac, Indomethacin, Magnesium Sulfate, Lidocaine, Pregabalin

Documented between 1 hour before anesthesia start to anesthesia end.

Delivered using one of the following routes: intravenous (IV), oral, or by enteric tube.

© 2024 Burns ML et al. *JAMA Network Open.*

Supplemental Material 7
Oral Morphine Equivalency (OME) Conversions

A normalized oral morphine equivalency (OME) value was calculated for each surgery by the following method:

1. Identify contributing opioid medications, route, and dose between anesthesia start and anesthesia end.
    a. Medications missing values essential to calculating the OME were flagged as missing OME.
2. Calculate an OME value for each administered opioid medication using the table below and sum the total OME for the surgery.
3. Normalize the OME value by dividing my patient weight (kg) and anesthesia duration (anesthesia end - anesthesia start, minutes)
    a. Surgeries missing patient weight and/or anesthesia start or end times were flagged as missing these values.
4. Surgeries with either of the two flags mentioned above were omitted from the study.

Additional information around the OME phenotype can be found here:

https://phenotypes.mpog.org/Oral%20Morphine%20Equivalent%20(Normalized)

Example: A 70 kg patient receives 100 mcg of fentanyl IV for induction for a 2.4-hour surgery. Here is the normalized OME for that fentanyl dose:

(1) First, we calculate the OME for the medication:

0.1mg IV fentanyl * (30 mg oral morphine/0.1mg IV fentanyl) = 30 OME

© 2024 Burns ML et al. *JAMA Network Open.*

(2) Next, we normalize the OME based on the patient's weight (kg) and the anesthesia duration from the surgery (hour):

30 OME / 70 kg / 2.4 hour = 0.18 mg OME/kg/hr

*If anesthesia duration <60 min, the calculation uses 1 hour.

The following conversions were used for oral morphine equivalency calculations:

Opioid	Route	Equivalence (mg)
Codeine[1,2]	Oral	200
Hydrocodone[2]	Oral	30
Hydromorphone[1,2]	Oral	7.5
Meperidine[1]	Oral	300
Methadone[2,8]	Oral	4 (1-20 mg/day),<br>8 (21-40 mg/day),<br>10 (41-60 mg/day),<br>12 (>60 mg/day)
Morphine[1,2]	Oral	30
MS Contin (controlled release morphine)[9]	Oral	30
Oxycodone[1,2]	Oral	20
Oxymorphone[1,2]	Oral	10
Tramadol[2]	Oral	300
Fentanyl[2]	Transdermal	72
Buprenorphine (Suboxone)[3]	Sublingual	75

© 2024 Burns ML et al. *JAMA Network Open.*

Alfentanil[7]	IV	0.5
Buprenorphine (Suboxone)[1]	IV	0.4
Butorphanol[1]	IV	2
Codeine[1]	IV	100
Fentanyl[1]	IV	0.1
Hydromorphone (Dilaudid)[1]	IV	1.5
Meperidine[1]	IV	100
Methadone[3]	IV	5
Morphine[1]	IV	10
Nalbuphine[1]	IV	10
Oxymorphone[1]	IV	1
Remifentanil	IV	0
Sufentanil[5]	IV	0.02
Tramadol[1]	IV	100
Fentanyl[6]	epidural	0.03
Hydromorphone (Dilaudid)[6]	epidural	0.3
Morphine[4]	epidural	1
Sufentanil[5,6]	epidural	0.006
Fentanyl[6]	IT	0.01
Hydromorphone (Dilaudid)[6]	IT	0.06
Meperidine	IT	0.1
Morphine[4]	IT	0.1
Sufentanil[6]	IT	0.003

eTable 4: Oral Morphine Equivalency (OME) Conversions.

© 2024 Burns ML et al. *JAMA Network Open.*

Opioid medications are listed along with route of administration and the relative equivalency in mg. This table was used to calculate the OME for each administered opioid medication.

1. APS. Principles of Analgesic Use, 7th Edition. In: Christopher M. Herndon PA, Beth Darnall, Craig Hartrick, Keith Hecht, Mary Lyons, Jahangir Maleki, Renee Manworren, Christine Miaskowski, Nalini Sehgal, editor. Principles of Analgesic Use. 7th ed2016. p. Table 2.

2. Dowell D HT, Chou R. CDC Guideline for Prescribing Opioids for Chronic Pain — United States, 2016. MMWR Recomm Rep 2016. 2016;65(No. RR-1):1–49.

3. McPherson, M. L. "Demystifying opioid conversion calculations." Bethesda, MD: American Society of Health-System Pharmacists (2009).

4. Krames, Elliot S. "Intrathecal infusional therapies for intractable pain: patient management guidelines." Journal of pain and symptom management 8, no. 1 (1993): 36-46.

5. Anderson, Robert, Joseph H. Saiers, Stephen Abram, and Christian Schlicht. "Accuracy in equianalgesic dosing: conversion dilemmas." Journal of pain and symptom management 21, no. 5 (2001): 397-406.

6. OpenAnesthesia , https://www.openanesthesia.org/, accessed 9/21/2020

7. Koyyalagunta, D. Pain Management, 2007, Chapter 113: Opioid Analgesics. Pain Management, 113(2), 2007, Pages 939-964

© 2024 Burns ML et al. *JAMA Network Open.*

8. Walker, Paul W., Shana Palla, Be-Lian Pei, Guddi Kaur, Karen Zhang, Jeanine Hanohano, Mark Munsell, and Eduardo Bruera. "Switching from methadone to a different opioid: what is the equianalgesic dose ratio?." Journal of palliative medicine 11, no. 8 (2008): 1103-1108.

9. https://www.healthcare.uiowa.edu/familymedicine/fpinfo/Docs/adultopioidrefguide.htm, accessed 8/12/2020

© 2024 Burns ML et al. *JAMA Network Open.*

Supplemental Material 8
Individual Hospitals Ranked by Intraoperative Opioid Administration

De-identified Institution	Mean (Rank)	Abdomen: Upper	Abdomen: Lower	Cardiac	Hysterectomy	Hip	Knee	Spine	Vascular
1	**0.1 (1)**	0.09 (1)	0.08 (1)	0.31 (9)	0.07 (1)	0.09 (3)	0.08 (6)	0.04 (1)	0.08 (1)
2	**0.16 (2)**	0.15 (2)	0.18 (3)	0.18 (2)	0.14 (3)	0.17 (14)	0.14 (14)	0.19 (7)	0.12 (3)
3	**0.16 (3)**	0.16 (3)	0.18 (2)	0.21 (3)	0.17 (4)	0.18 (16)	0.14 (13)	0.11 (2)	0.14 (4)
4	**0.18 (4)**	0.22 (7)	0.25 (8)	0.24 (4)	0.18 (6)	0.11 (6)	0.12 (11)	0.18 (5)	0.17 (7)
5	**0.19 (5)**	0.19 (5)	0.19 (4)	0.39 (15)	0.07 (2)	0.11 (5)	0.1 (7)	0.23 (12)	0.23 (23)
6	**0.2 (6)**	0.22 (6)	0.24 (7)	0.35 (10)	0.19 (11)	0.07 (2)	0.05 (2)	0.26 (23)	0.2 (14)
7	**0.2 (7)**	0.18 (4)	0.21 (5)	0.54 (36)	0.19 (7)	0.1 (4)	0.08 (5)	0.17 (4)	0.15 (6)
8	**0.21 (8)**	0.24 (12)	0.25 (9)	-	0.2 (13)	0.17 (13)	0.17 (22)	0.2 (9)	0.21 (15)
9	**0.21 (9)**	0.25 (13)	0.29 (14)	-	0.18 (5)	0.18 (17)	0.15 (17)	0.23 (14)	-
10	**0.23 (10)**	0.24 (10)	0.27 (12)	0.4 (16)	0.19 (10)	0.21 (25)	0.16 (19)	0.2 (8)	0.15 (5)
11	**0.23 (11)**	0.28 (17)	0.3 (16)	0.52 (34)	0.25 (16)	0.06 (1)	0.05 (1)	0.17 (3)	0.2 (13)
12	**0.23 (12)**	0.3 (20)	0.33 (25)	0.07 (1)	0.28 (25)	0.24 (31)	0.24 (39)	0.24 (15)	0.18 (10)
13	**0.24 (13)**	0.24 (11)	0.26 (10)	0.3 (8)	0.19 (12)	0.24 (34)	0.2 (31)	0.25 (21)	0.19 (11)
14	**0.24 (14)**	0.23 (9)	0.28 (13)	0.47 (27)	0.19 (9)	0.19 (22)	0.15 (15)	0.23 (13)	0.17 (8)
15	**0.25 (15)**	0.29 (19)	0.32 (23)	-	0.31 (29)	0.22 (27)	0.23 (37)	0.27 (25)	0.12 (2)
16	**0.25 (16)**	0.27 (15)	0.26 (11)	0.39 (14)	0.23 (14)	0.24 (32)	0.19 (29)	0.25 (20)	0.22 (19)
17	**0.25 (17)**	0.38 (38)	0.36 (34)	0.24 (5)	0.27 (20)	0.13 (7)	0.14 (12)	0.28 (26)	0.23 (25)
18	**0.26 (18)**	0.25 (14)	0.29 (15)	0.46 (25)	0.24 (15)	0.21 (26)	0.18 (26)	0.24 (16)	0.21 (18)
19	**0.26 (19)**	0.31 (23)	0.35 (30)	0.36 (12)	0.28 (24)	0.17 (12)	0.12 (10)	0.25 (19)	0.28 (34)
20	**0.27 (20)**	0.33 (27)	0.33 (24)	0.4 (17)	0.28 (26)	0.16 (11)	0.15 (16)	0.25 (17)	0.23 (24)
21	**0.27 (21)**	0.22 (8)	0.23 (6)	0.43 (21)	0.19 (8)	0.24 (33)	0.2 (30)	0.31 (36)	0.3 (37)
22	**0.27 (22)**	0.32 (25)	0.31 (18)	0.27 (6)	0.26 (17)	0.32 (44)	0.28 (43)	0.18 (6)	0.25 (27)
23	**0.28 (23)**	0.37 (36)	0.41 (38)	0.42 (20)	0.33 (34)	0.15 (10)	0.07 (4)	0.29 (31)	0.21 (16)
24	**0.28 (24)**	0.28 (16)	0.32 (19)	0.49 (28)	0.26 (18)	0.26 (36)	0.18 (28)	0.28 (30)	0.18 (9)
25	**0.28 (25)**	0.34 (30)	0.34 (28)	0.53 (35)	0.28 (23)	0.15 (9)	0.11 (9)	0.26 (22)	0.25 (28)
26	**0.28 (26)**	0.31 (21)	0.32 (21)	0.59 (39)	0.28 (22)	0.27 (40)	0.07 (3)	0.25 (18)	0.19 (12)
27	**0.29 (27)**	0.35 (32)	0.35 (29)	0.43 (22)	0.34 (37)	0.2 (24)	0.16 (20)	0.28 (27)	0.22 (20)
28	**0.29 (28)**	0.34 (29)	0.34 (27)	-	0.31 (30)	0.29 (41)	0.25 (42)	0.22 (11)	-
29	**0.29 (29)**	0.33 (28)	0.3 (17)	0.42 (19)	0.26 (19)	0.25 (35)	0.24 (40)	0.3 (34)	0.23 (26)
30	**0.3 (30)**	0.31 (22)	0.32 (20)	0.45 (23)	0.27 (21)	0.23 (29)	0.21 (32)	0.28 (29)	0.3 (36)
31	**0.25 (31)**	0.4 (41)	0.41 (39)	0.28 (7)	0.41 (43)	0.19 (19)	0.18 (27)	0.28 (28)	0.21 (17)
32	**0.3 (32)**	0.28 (18)	0.32 (22)	0.47 (26)	0.32 (31)	0.19 (21)	0.22 (36)	0.37 (43)	0.22 (22)
33	**0.3 (33)**	0.37 (35)	0.39 (37)	0.35 (11)	0.34 (38)	0.18 (15)	0.17 (21)	0.26 (24)	0.36 (41)
34	**0.31 (34)**	0.36 (33)	0.36 (32)	0.6 (40)	0.32 (33)	0.14 (8)	0.11 (8)	0.3 (33)	0.29 (35)
35	**0.31 (35)**	0.32 (26)	0.33 (26)	0.52 (32)	0.29 (27)	0.26 (38)	0.18 (25)	0.31 (35)	0.31 (40)
36	**0.32 (36)**	0.37 (34)	0.36 (33)	0.41 (18)	0.3 (28)	0.26 (39)	0.22 (35)	0.38 (44)	0.22 (21)
37	**0.32 (37)**	0.39 (39)	0.42 (40)	0.46 (24)	0.34 (36)	0.2 (23)	0.17 (23)	0.32 (38)	0.26 (29)
38	**0.32 (38)**	0.35 (31)	0.43 (42)	0.52 (33)	0.34 (39)	0.19 (20)	0.17 (24)	0.33 (41)	0.26 (31)
39	**0.32 (39)**	0.43 (44)	0.45 (45)	-	0.4 (41)	0.18 (18)	0.16 (18)	0.32 (40)	-
40	**0.33 (40)**	0.31 (24)	0.35 (31)	0.57 (38)	0.33 (35)	0.3 (43)	0.32 (46)	0.21 (10)	0.26 (30)
41	**0.33 (41)**	0.37 (37)	0.37 (35)	0.49 (30)	0.32 (32)	0.26 (37)	0.21 (33)	0.32 (39)	0.3 (39)
42	**0.36 (42)**	0.4 (42)	0.42 (41)	0.38 (13)	0.42 (44)	0.37 (46)	0.29 (44)	0.29 (32)	0.27 (33)
43	**0.36 (43)**	0.39 (40)	0.38 (36)	0.49 (29)	0.38 (40)	0.33 (45)	0.3 (45)	0.32 (37)	0.27 (32)
44	**0.37 (44)**	0.44 (45)	0.45 (44)	0.5 (31)	0.41 (42)	0.23 (30)	0.22 (34)	0.39 (45)	0.3 (38)
45	**0.39 (45)**	0.42 (43)	0.43 (43)	0.55 (37)	0.42 (45)	0.29 (42)	0.24 (41)	0.34 (42)	0.38 (43)
46	**0.4 (46)**	0.52 (46)	0.55 (46)	-	0.47 (46)	0.23 (28)	0.23 (38)	0.43 (46)	0.37 (42)
AVG	0.27	0.31	0.32	0.41	0.28	0.20	0.17	0.26	0.23
STDEV	0.06	0.09	0.08	0.12	0.09	0.07	0.07	0.07	0.07

eFigure 2: Individual hospitals ranked by intraoperative opioid administration
Values are unadjusted intraoperative opioid administration means (OME/kg/hr), with each row representing a de-identified hospital. The mean across the hospital is listed overall (Mean) and by procedure, with the column rank in parentheses. Heat map color is determined within each

© 2024 Burns ML et al. *JAMA Network Open.*

column. Rows are ordered by opioid administration with the lowest (top) to highest (bottom) institutional mean across 8 surgical categories (upper abdomen, lower abdomen, cardiac, hysterectomy, orthopedic hip, orthopedic knee, orthopedic spine, and vascular), each separately represented as columns in the table. Dash (-) indicates there were no surgeries at the institution within the specified surgical category.

© 2024 Burns ML et al. *JAMA Network Open.*

Supplemental Material 9
Anesthesiologist, Surgeon, and Hospital Variation by Surgical Category

Graphs show intraoperative opioid administration (OME/kg/hr) by individual anesthesiologist or surgeon, plotted by increasing mean. Bars represent 95% CI.

Anesthesiologist

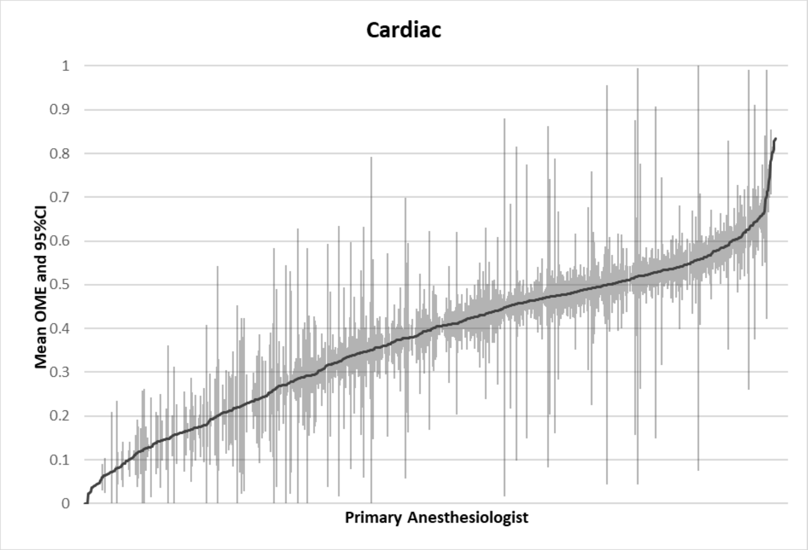

eFigure 3: Intraoperative opioid administration by anesthesiologist for cardiac surgeries.

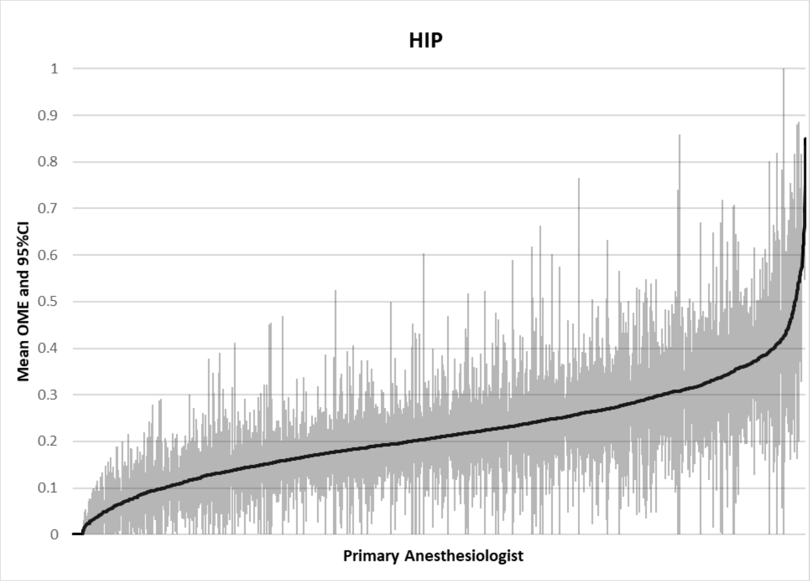

© 2024 Burns ML et al. *JAMA Network Open.*

eFigure 4: Intraoperative opioid administration by anesthesiologist for hip surgeries.

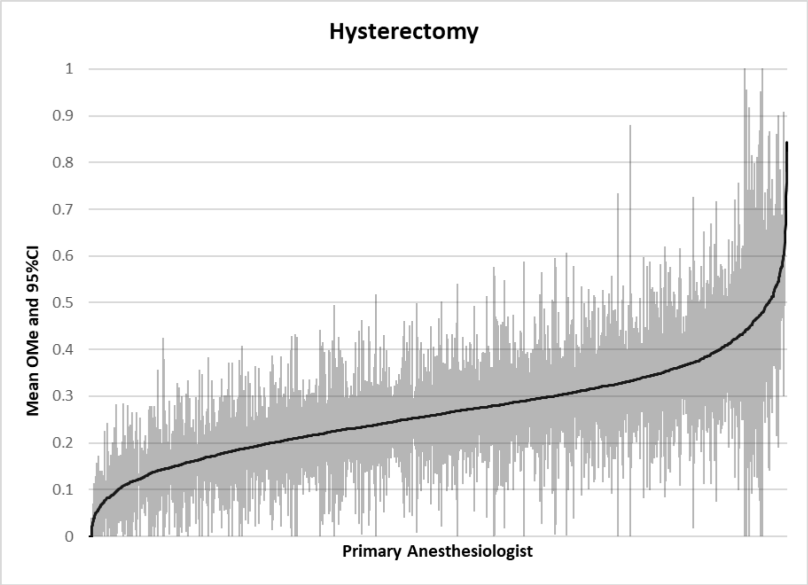

eFigure 5: Intraoperative opioid administration by anesthesiologist for hysterectomy surgeries.

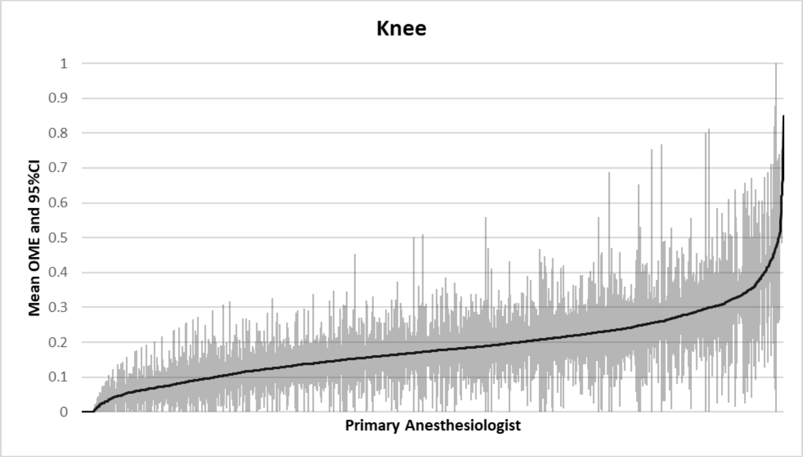

eFigure 6: Intraoperative opioid administration by anesthesiologist for knee surgeries.

© 2024 Burns ML et al. *JAMA Network Open.*

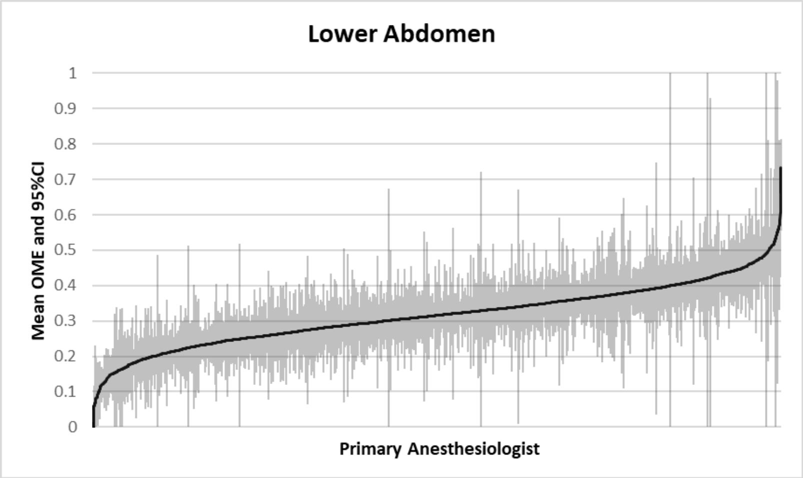

eFigure 7: Intraoperative opioid administration by anesthesiologist for lower abdominal surgeries.

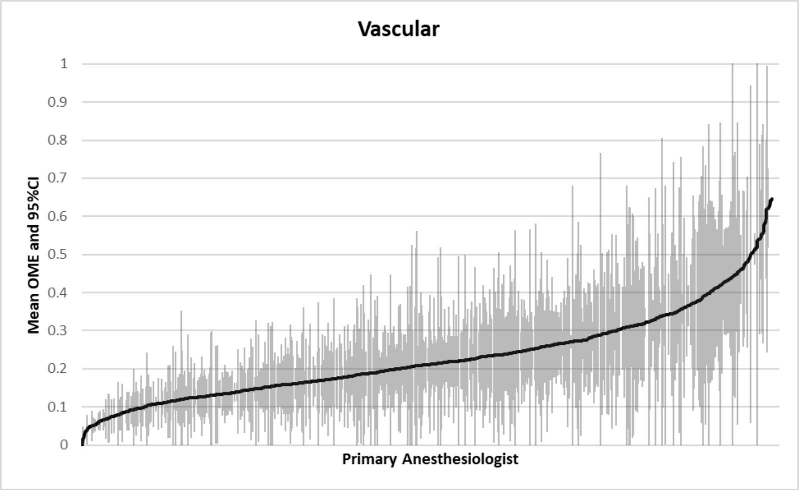

eFigure 8: Intraoperative opioid administration by anesthesiologist for vascular surgeries.

© 2024 Burns ML et al. *JAMA Network Open.*

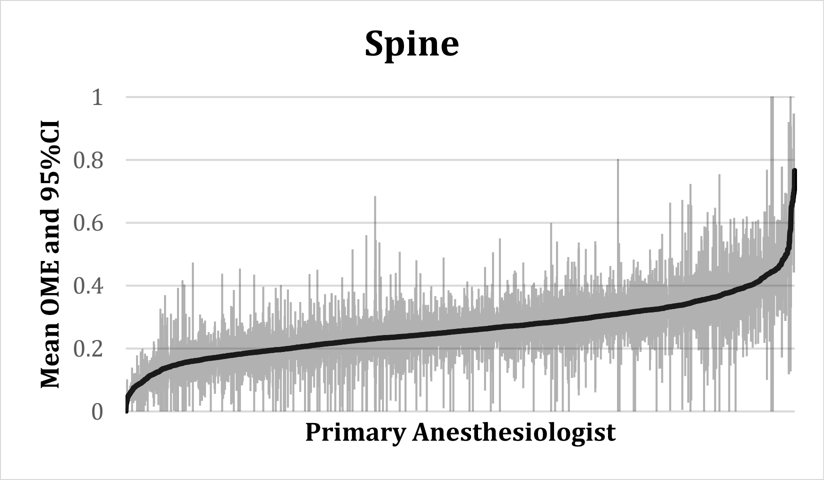

eFigure 9: Intraoperative opioid administration by anesthesiologist for spine surgeries.

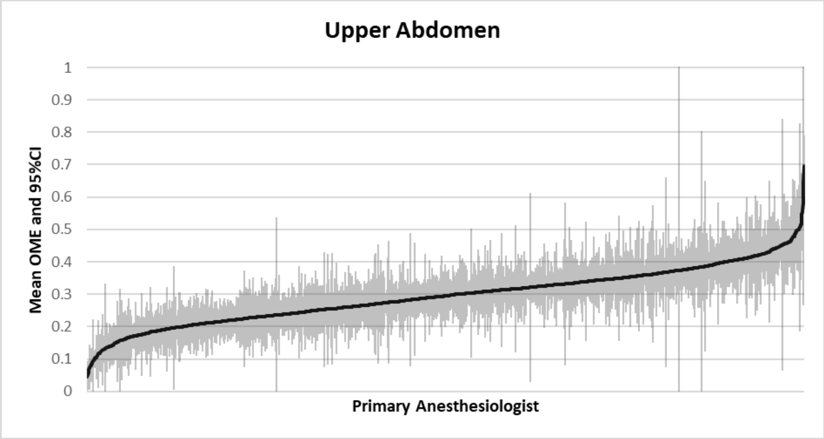

eFigure 10: Intraoperative opioid administration by anesthesiologist for upper abdominal surgeries.

Surgeon

© 2024 Burns ML et al. *JAMA Network Open.*

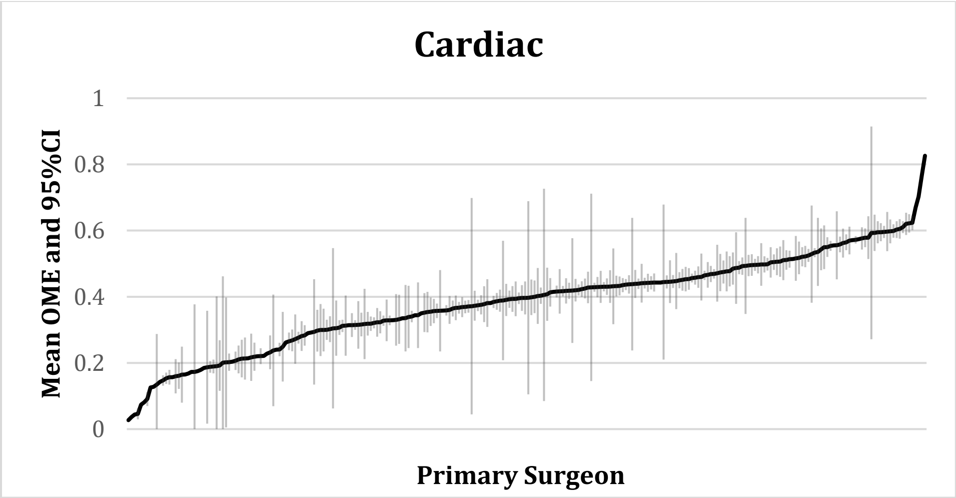

eFigure 11: Intraoperative opioid administration by surgeon for cardiac surgeries.

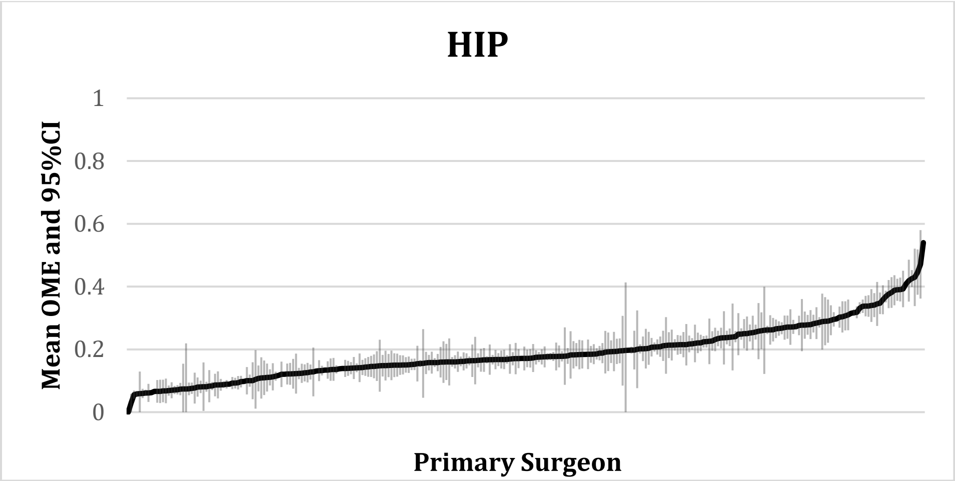

eFigure 12: Intraoperative opioid administration by surgeon for hip surgeries.

© 2024 Burns ML et al. *JAMA Network Open.*

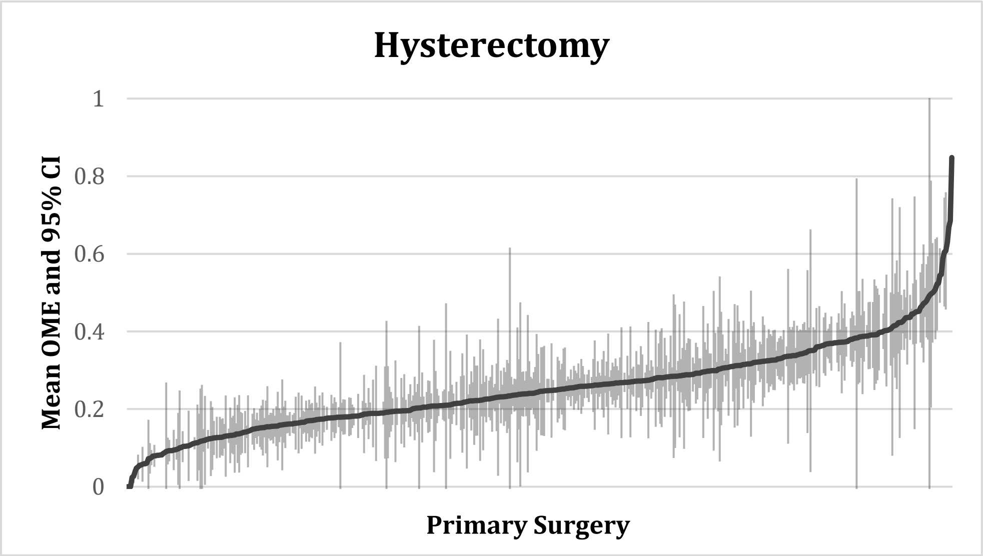

eFigure 13: Intraoperative opioid administration by surgeon for hysterectomy surgeries.

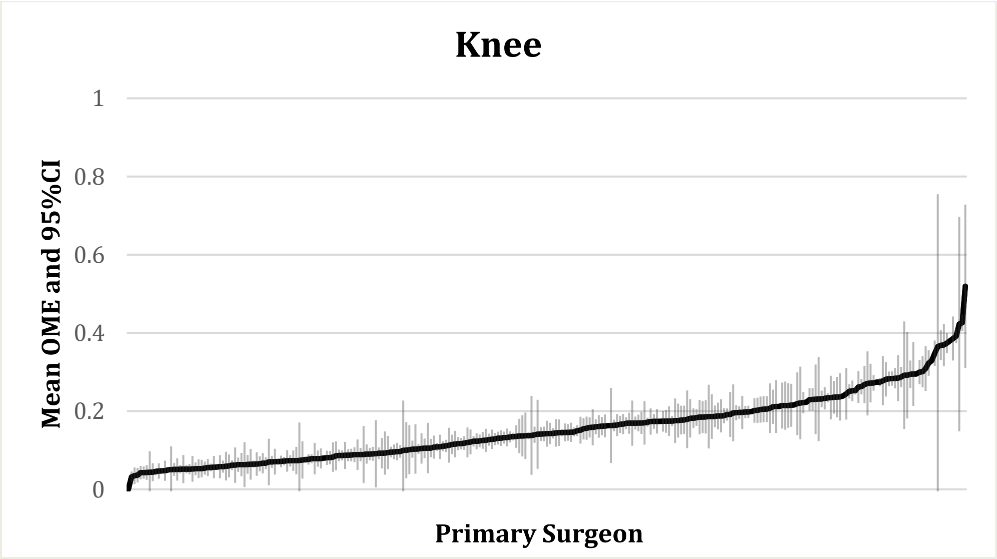

eFigure 14: Intraoperative opioid administration by surgeon for knee surgeries.

© 2024 Burns ML et al. *JAMA Network Open.*

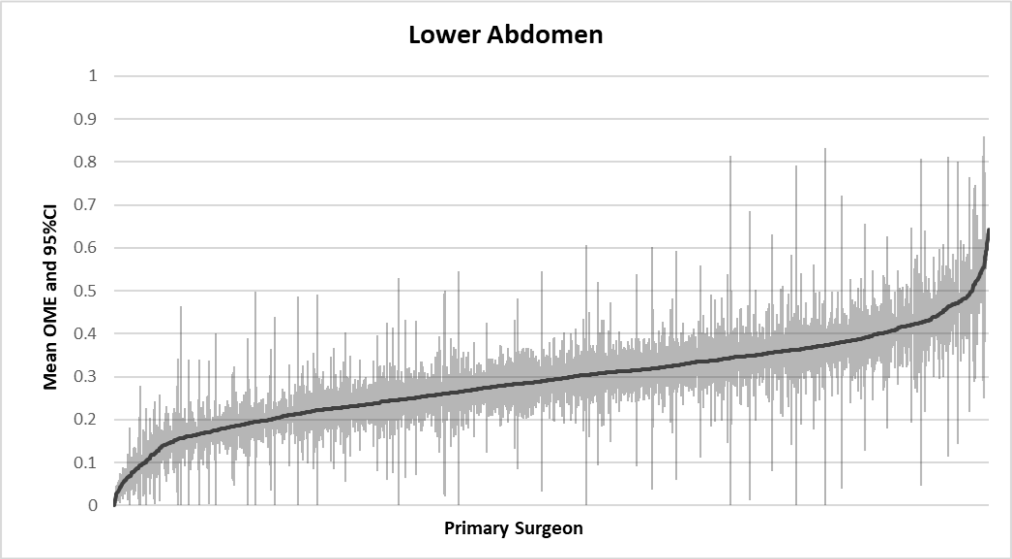

eFigure 15: Intraoperative opioid administration by surgeon for lower abdominal surgeries.

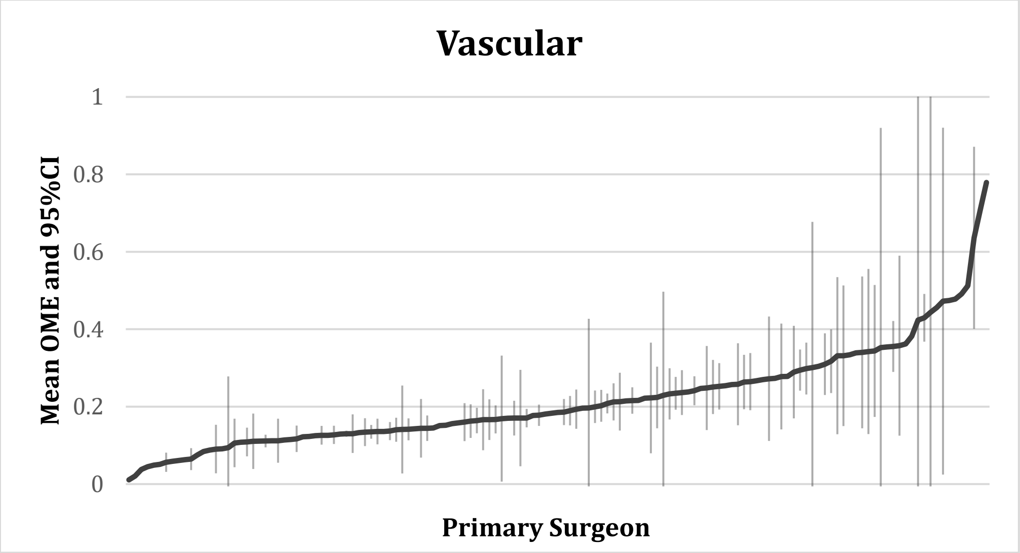

eFigure 16: Intraoperative opioid administration by surgeon for vascular surgeries.

© 2024 Burns ML et al. *JAMA Network Open.*

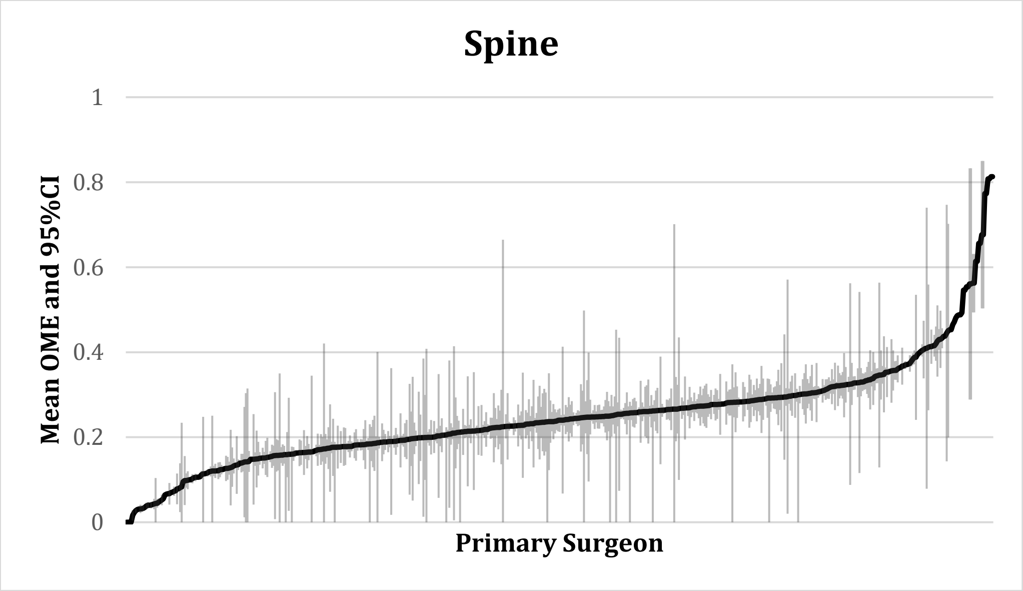

eFigure 17: Intraoperative opioid administration by surgeon for spine surgeries.

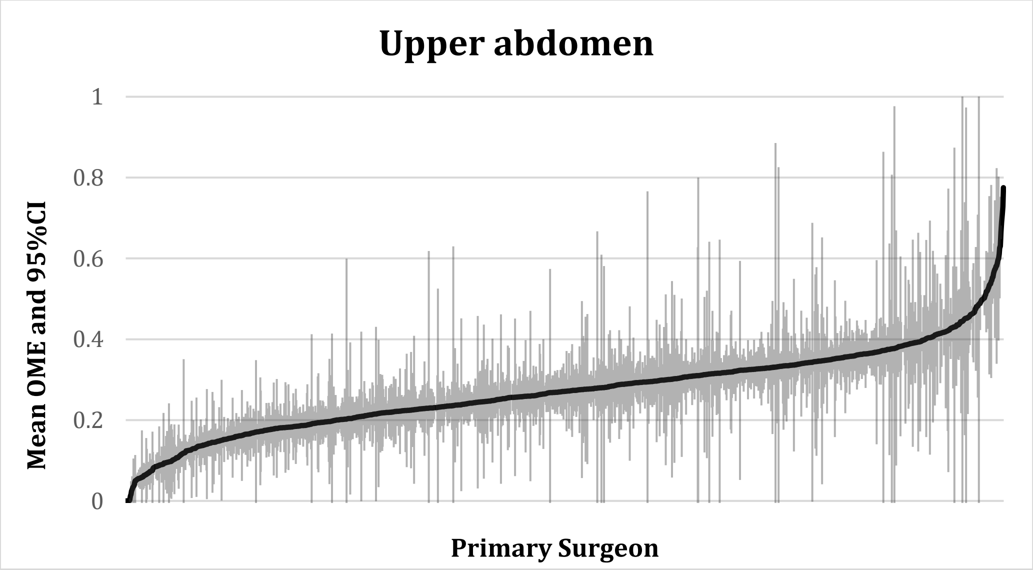

eFigure 18: Intraoperative opioid administration by surgeon for upper abdominal surgeries.

Hospital

© 2024 Burns ML et al. *JAMA Network Open.*

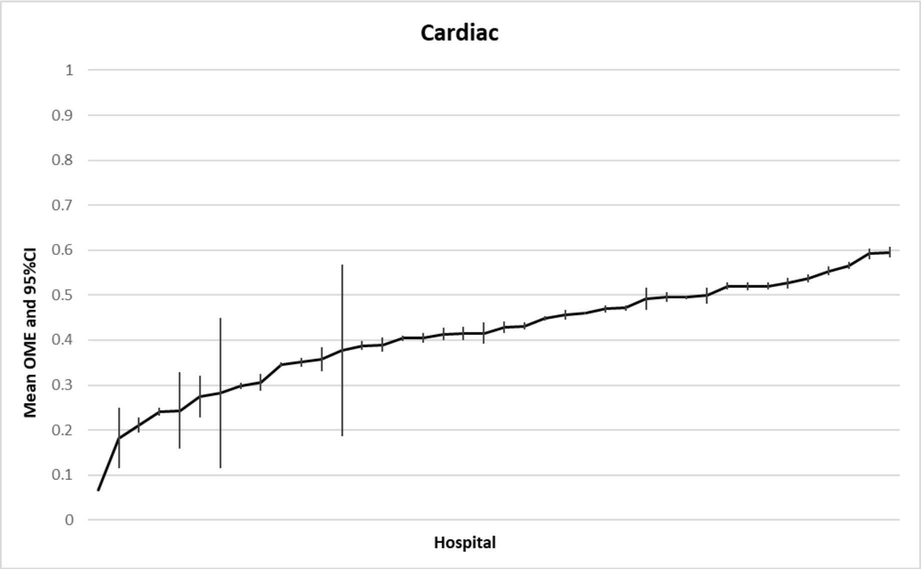

eFigure 19: Intraoperative opioid administration by hospital for cardiac surgeries.

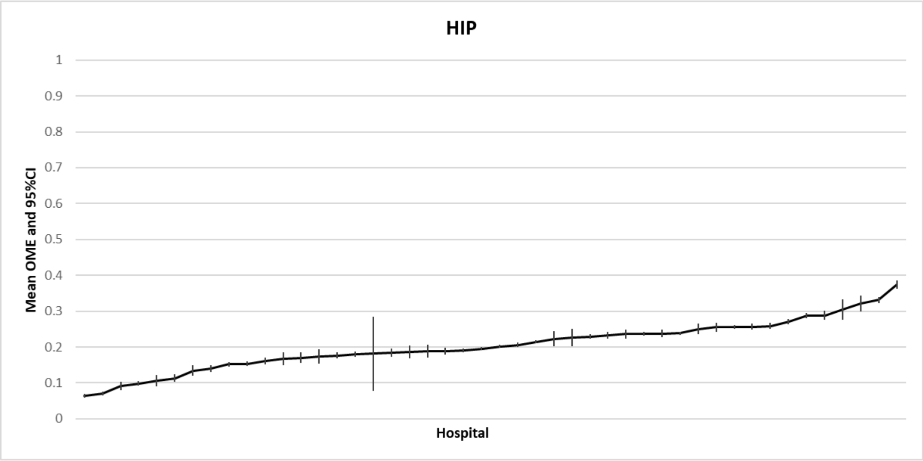

eFigure 20: Intraoperative opioid administration by hospital for hip surgeries.

© 2024 Burns ML et al. *JAMA Network Open.*

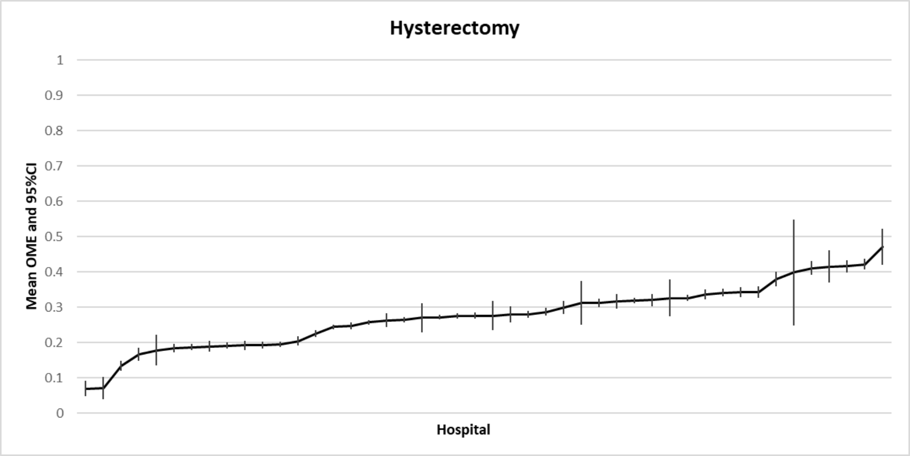

eFigure 21: Intraoperative opioid administration by hospital for hysterectomy surgeries.

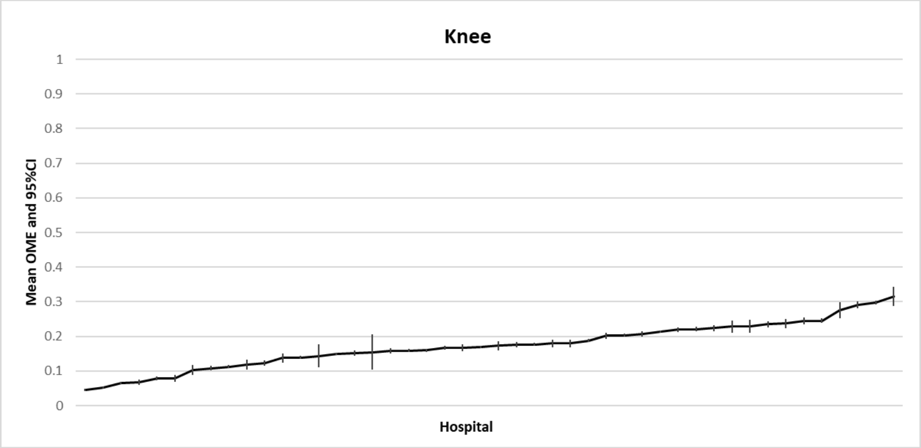

eFigure 22: Intraoperative opioid administration by hospital for knee surgeries.

© 2024 Burns ML et al. *JAMA Network Open.*

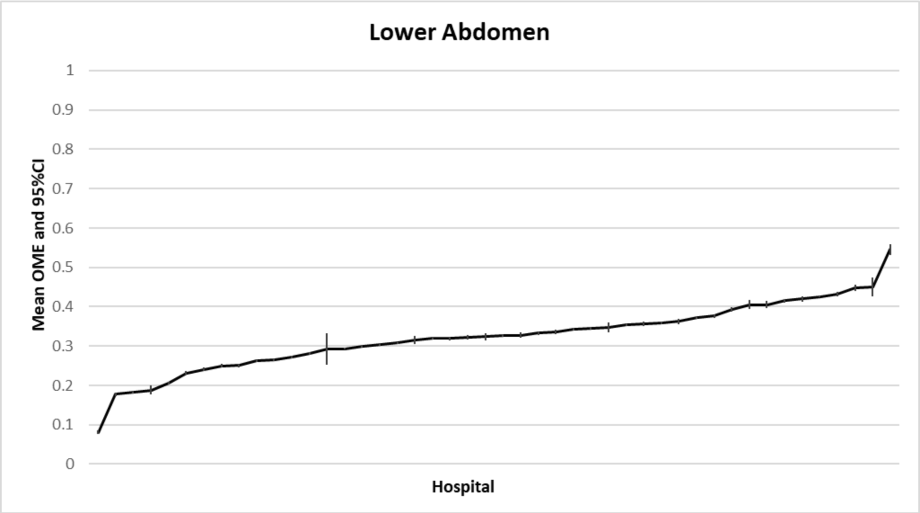

eFigure 23: Intraoperative opioid administration by hospital for lower abdominal surgeries.

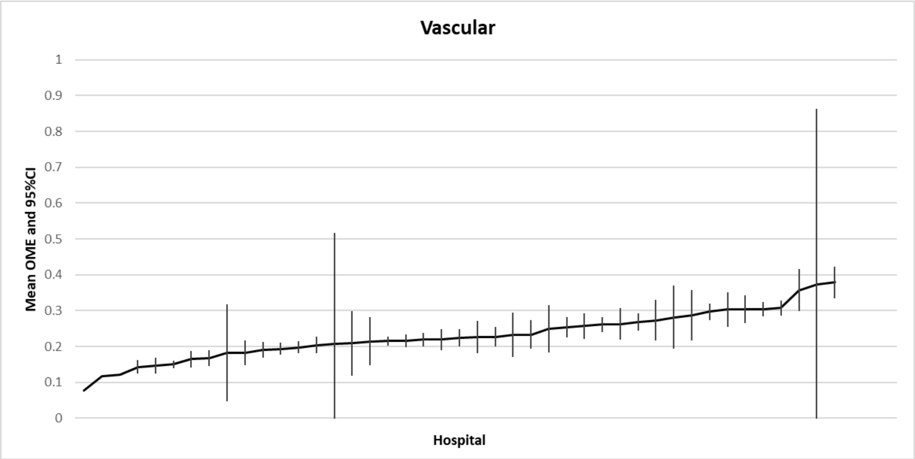

eFigure 24: Intraoperative opioid administration by hospital for vascular surgeries.

© 2024 Burns ML et al. *JAMA Network Open.*

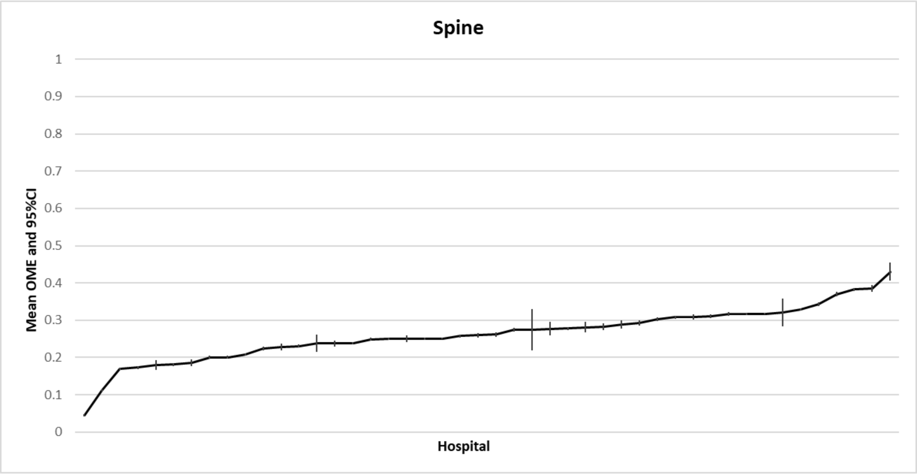

eFigure 25: Intraoperative opioid administration by hospital for spine surgeries.

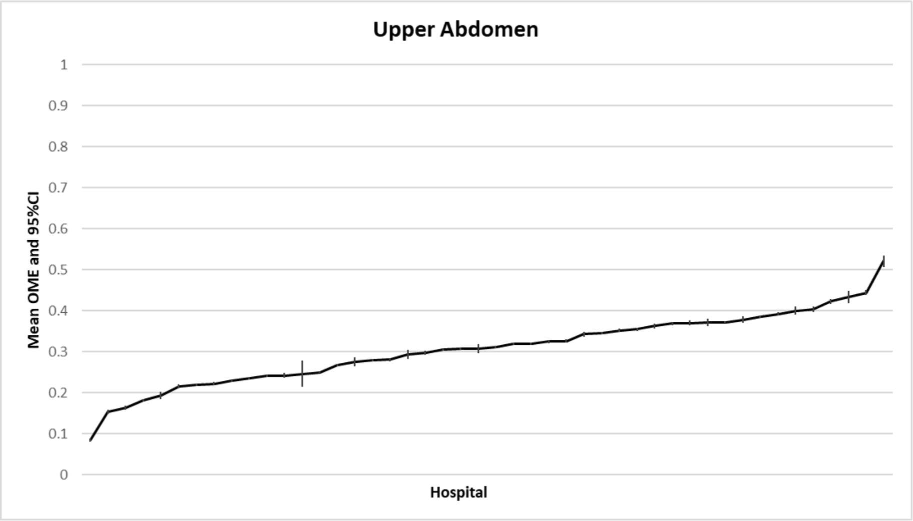

eFigure 26: Intraoperative opioid administration by hospital for upper abdominal surgeries.

© 2024 Burns ML et al. *JAMA Network Open.*

Supplemental Material 10
Full Model Results for GLMM with Random Intercept

GLMM with random intercept for Anesthesiologist and Hospital				GLMM with random intercept for Surgeon and Hospital			
		Full Sample				Full Sample	
Effect	Level	Estimate (95%CI)	p-value	Effect	Level	Estimate (95%CI)	p-value
Intercept		0.477(0.457,0.497)	0	Intercept		0.403(0.381,0.425)	0
agecat1	2	-0.03(-0.031,-0.028)	<.0001	agecat1	2	-0.023(-0.024,-0.021)	<.0001
agecat1	3	-0.052(-0.053,-0.05)	<.0001	agecat1	3	-0.034(-0.036,-0.033)	<.0001
agecat1	4	-0.072(-0.073,-0.071)	<.0001	agecat1	4	-0.046(-0.048,-0.045)	<.0001
agecat1	5	-0.095(-0.097,-0.094)	<.0001	agecat1	5	-0.064(-0.066,-0.062)	<.0001
agecat1	1	ref		agecat1	1	ref	
BMI_WHO1	Class I Obesity	-0.071(-0.072,-0.07)	<.0001	BMI_WHO1	Class I Obesity	-0.069(-0.071,-0.068)	<.0001
BMI_WHO1	Class II Obesity	-0.1(-0.101,-0.098)	<.0001	BMI_WHO1	Class II Obesity	-0.094(-0.096,-0.092)	<.0001
BMI_WHO1	Class III Obesity	-0.142(-0.144,-0.141)	<.0001	BMI_WHO1	Class III Obesity	-0.135(-0.137,-0.133)	<.0001
BMI_WHO1	Overweight	-0.039(-0.04,-0.039)	<.0001	BMI_WHO1	Overweight	-0.039(-0.04,-0.038)	<.0001
BMI_WHO1	Nomral or Underweight	ref		BMI_WHO1	Nomral or Underweight	ref	
race_cat	NH Black	-0.011(-0.013,-0.01)	<.0001	race_cat	NH Black	-0.005(-0.007,-0.004)	<.0001
race_cat	Other	0.005(0.004,0.006)	<.0001	race_cat	Other	0.004(0.003,0.006)	<.0001
race_cat	NH White	ref		race_cat	NH White	ref	
Gender	Female	0.026(0.025,0.027)	<.0001	Gender	Female	0.028(0.027,0.029)	<.0001
Gender	Unknown	0.015(-0.013,0.044)	0.2951	Gender	Unknown	0.018(-0.014,0.049)	0.2685
Gender	Male	ref		Gender	Male	ref	
ASA_Class	ASA Class 1	-0.042(-0.045,-0.04)	<.0001	ASA_Class	ASA Class 1	0.013(0.01,0.016)	<.0001
ASA_Class	ASA Class 2	-0.051(-0.053,-0.05)	<.0001	ASA_Class	ASA Class 2	0.007(0.005,0.01)	<.0001
ASA_Class	ASA Class 3	-0.046(-0.048,-0.045)	<.0001	ASA_Class	ASA Class 3	0.007(0.005,0.009)	<.0001
ASA_Class	ASA Class 4	ref		ASA_Class	ASA Class 4	ref	
Emergency_Status_rc	Unmapped	0.021(0.013,0.03)	<.0001	Emergency_Status_rc	Unmapped	0.009(0.001,0.018)	0.0364
Emergency_Status_rc	Yes	0.016(0.014,0.017)	<.0001	Emergency_Status_rc	Yes	0.005(0.003,0.007)	<.0001
Emergency_Status_rc	No	ref		Emergency_Status_rc	No	ref	
Length_of_Stay		0.014(0.012,0)	<.0001	Length_of_Stay		-0.001(-0.008,0)	0.757
Case_Duration		-0.023(-0.023,0)	<.0001	Case_Duration		-0.037(-0.038,0)	<.0001
Weekend	Weekday	-0.017(-0.019,-0.016)	<.0001	Weekend	Weekday	-0.011(-0.013,-0.009)	<.0001
Weekend	Weekend	ref		Weekend	Weekend	ref	
Holiday	Yes	0.003(-0.002,0.009)	0.2657	Holiday	Yes	-0.001(-0.008,0.007)	0.8684
Holiday	No	ref		Holiday	No	ref	
AidsHIV		0.007(0,0.015)	0.0636	AidsHIV		0.01(0,0.02)	0.0463
AlcoholAbuse		ref		AlcoholAbuse		ref	

© 2024 Burns ML et al. *JAMA Network Open.*

BloodLossAnemia	-0.01(-0.013,-0.007)	<.0001
CardiacArrhythmias	0.003(0.002,0.004)	<.0001
ChronicPulmonary Dise	-0.001(-0.002,0)	0.1934
Coagulopathy	0.001(0,0.003)	0.0702
CongestiveHeartFailu	-0.003(-0.005,-0.002)	0.0002
DeficiencyAnemia	-0.005(-0.007,-0.003)	<.0001
Depression	-0.003(-0.004,-0.002)	<.0001
DiabetesComplicated	-0.002(-0.005,0)	0.0245
DiabetesUncomplicate	0.002(0.001,0.003)	<.0001
DrugAbuse	0.019(0.016,0.021)	<.0001
FluidElectrolyteDiso	0.01(0.009,0.012)	<.0001
HypertensionComplica	-0.002(-0.004,0)	0.1109
HypertensionUncompli	-0.001(-0.002,-0.001)	0.0009
Hypothyroidism	-0.009(-0.01,-0.008)	<.0001
LiverDisease	-0.007(-0.008,-0.005)	<.0001
Lymphoma	-0.002(-0.005,0.002)	0.4567
MetastaticCancer	0.006(0.004,0.007)	<.0001
Obesity	-0.018(-0.019,-0.017)	<.0001
OtherNeurologicalDis	-0.014(-0.015,-0.012)	<.0001
Paralysis	-0.022(-0.025,-0.019)	<.0001
PepticUlcerDiseaseEx	0.006(0.002,0.009)	0.0021
PeripheralVascularDi	0.002(0.001,0.004)	0.0046
Psychoses	-0.011(-0.016,-0.007)	<.0001
PulmonaryCirculation	-0.013(-0.015,-0.011)	<.0001
RenalFailure	-0.016(-0.018,-0.015)	<.0001
RheumatoidArthritisC	-0.002(-0.004,0)	0.051
SolidTumorWithoutMet	-0.007(-0.008,-0.006)	<.0001
ValvularDisease	0.046(0.044,0.047)	<.0001
WeightLoss	-0.012(-0.013,-0.01)	<.0001

BloodLossAnemia	-0.004(-0.008,0)	0.0803
CardiacArrhythmias	-0.001(-0.003,0)	0.0511
ChronicPulmonary Dise	0.002(0.001,0.004)	0.0004
Coagulopathy	-0.005(-0.007,-0.002)	<.0001
CongestiveHeartFailu	-0.011(-0.014,-0.009)	<.0001
DeficiencyAnemia	-0.003(-0.006,0)	0.0295
Depression	0.001(0,0.002)	0.1063
DiabetesComplicated	-0.003(-0.006,0)	0.0647
DiabetesUncomplicate	0(-0.002,0.001)	0.7369
DrugAbuse	0.024(0.021,0.027)	<.0001
FluidElectrolyteDiso	0.001(-0.001,0.002)	0.2527
HypertensionComplica	0(-0.003,0.002)	0.8806
HypertensionUncompli	0.001(0,0.002)	0.09
Hypothyroidism	-0.006(-0.008,-0.005)	<.0001
LiverDisease	-0.004(-0.007,-0.002)	<.0001
Lymphoma	0.002(-0.003,0.007)	0.4801
MetastaticCancer	0.011(0.008,0.013)	<.0001
Obesity	-0.011(-0.013,-0.01)	<.0001
OtherNeurologicalDis	-0.007(-0.01,-0.005)	<.0001
Paralysis	-0.011(-0.015,-0.007)	<.0001
PepticUlcerDiseaseEx	0.008(0.003,0.013)	0.0007
PeripheralVascularDi	0(-0.002,0.002)	0.8436
Psychoses	-0.009(-0.015,-0.003)	0.0055
PulmonaryCirculation	-0.007(-0.01,-0.005)	<.0001
RenalFailure	-0.01(-0.013,-0.008)	<.0001
RheumatoidArthritisC	0.008(0.005,0.01)	<.0001
SolidTumorWithoutMet	-0.006(-0.008,-0.005)	<.0001
ValvularDisease	0.004(0.002,0.006)	0.0006
WeightLoss	0.007(0.005,0.009)	<.0001

Descr	Value
-2 Res Log Likelihood	-741609
AIC (Smaller is Better)	-741603
AICC (Smaller is Better)	-741603
BIC (Smaller is Better)	-741597

Descr	Value
-2 Res Log Likelihood	-741609
AIC (Smaller is Better)	-741603
AICC (Smaller is Better)	-741603
BIC (Smaller is Better)	-741597

© 2024 Burns ML et al. *JAMA Network Open.*

eTable 5: Full Model Results for generalized linear mixed model with random intercept. Results to the left are for the model for anesthesiologist and hospital; while results to the right are for the model for surgeon and hospital. GLMM = generalized linear mixed model.

© 2024 Burns ML et al. *JAMA Network Open.*

Supplemental Material 11
Adjusted Intra-class Correlation Coefficients by Surgical Group or Analgesic Category

Intraclass correlation coefficients (ICCs) of adjusted intraoperative opioid administration displayed by analgesic category and patient, provider (anesthesiologist or surgeon), and institution.

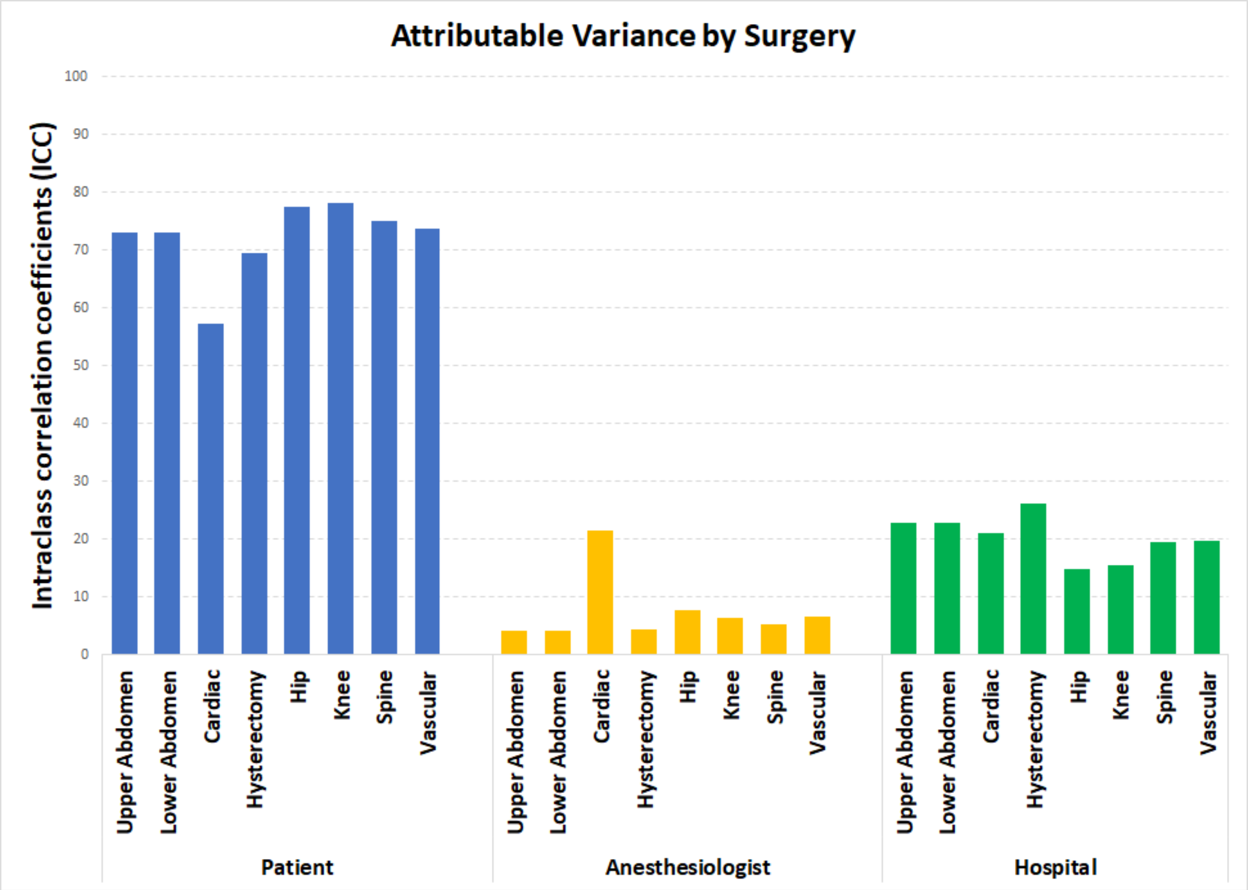

eFigure 27: Intraclass correlation coefficients (ICCs) of adjusted intraoperative opioid administration displayed by surgical category and patient, anesthesiologist, and hospital.

© 2024 Burns ML et al. *JAMA Network Open.*

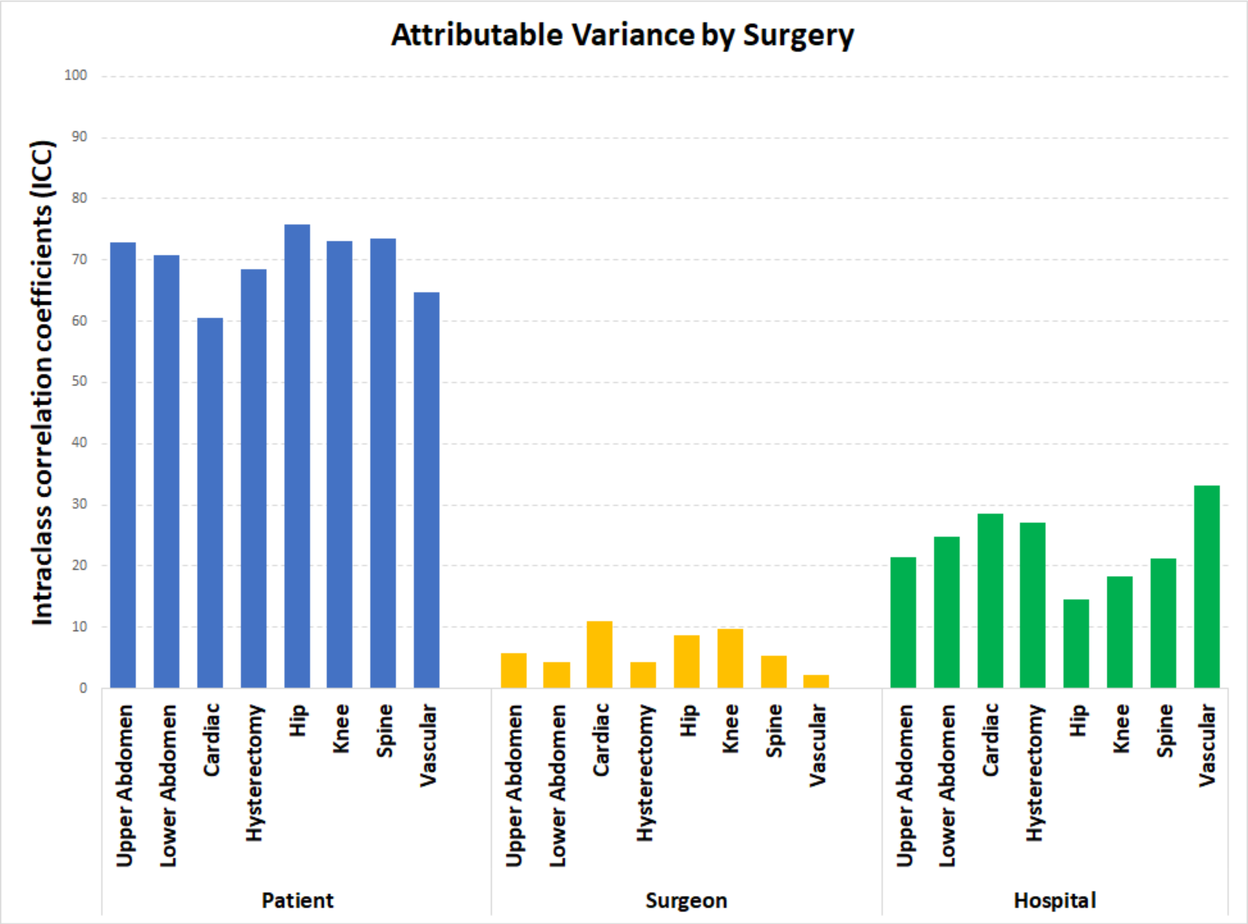

eFigure 28: Intraclass correlation coefficients (ICCs) of adjusted intraoperative opioid administration displayed by surgical category and patient, surgeon, and hospital.

© 2024 Burns ML et al. *JAMA Network Open.*

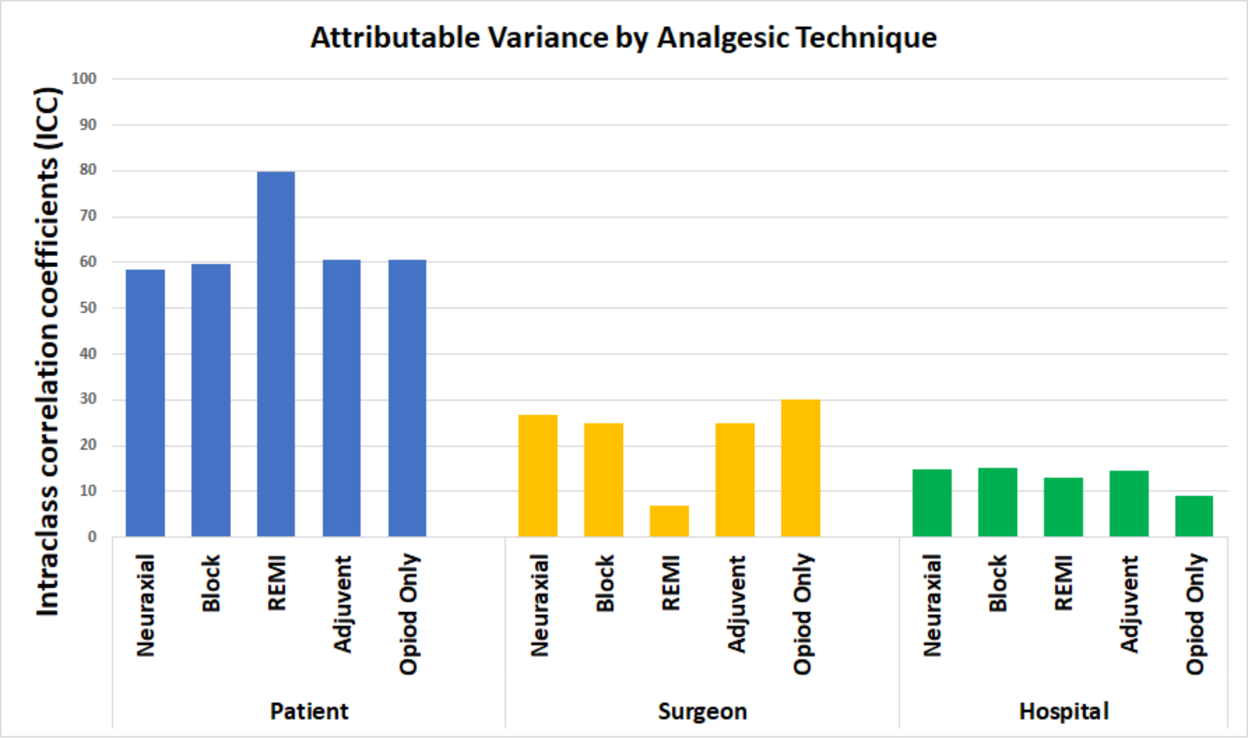

eFigure 29: Intraclass correlation coefficients (ICCs) of adjusted intraoperative opioid administration displayed by analgesic category and patient, surgeon, and hospital.

© 2024 Burns ML et al. *JAMA Network Open.*
