# Supplementary material for: Variation in Intraoperative Opioid Administration by Patient, Clinician, and Hospital Contribution
Source: JAMA Netw Open. 2024 Jan 16;7(1):e2351689. doi: 10.1001/jamanetworkopen.2023.51689 (PMC10792468; doi:10.1001/jamanetworkopen.2023.51689)
Supplement: Supplement 2. — Data Sharing Statement [file jamanetwopen-e2351689-s002.pdf]

Data Sharing Statement

Burns. Variation in Intraoperative Opioid Administration by Patient, Clinician, and Hospital Contribution. *JAMA Netw Open*. Published online January 16, 2024. doi:10.1001/jamanetworkopen.2023.51689

Data
Data available: No

Additional Information
Explanation for why data not available: The data used in this study is part of the MPOG collaborative and contains sensitive patient information.
